# IgM cleavage by *Streptococcus suis* reduces IgM bound to the bacterial surface and is a novel complement evasion mechanism

**DOI:** 10.1080/21505594.2018.1496778

**Published:** 2018-08-28

**Authors:** Viktoria Rungelrath, Christine Weiße, Nicole Schütze, Uwe Müller, Marita Meurer, Manfred Rohde, Jana Seele, Peter Valentin-Weigand, Michael Kirschfink, Andreas Beineke, Wieland Schrödl, René Bergmann, Christoph Georg Baums

**Affiliations:** a Institute for Bacteriology and Mycology, Centre for Infectious Diseases, Veterinary Faculty, University of Leipzig, Leipzig, Germany; b Institute of Immunology, Centre for Infectious Diseases, Veterinary Faculty, University of Leipzig, Leipzig, Germany; c Department of Physiological Chemistry and Research Center for Emerging Infections and Zoonoses (RIZ), University of Veterinary Medicine Hannover, Hannover, Germany; d Central Facility for Microscopy, Helmholtz Centre for Infection Research, Braunschweig, Germany; e Department of Neuropathology, University Medical Center Göttingen, Georg-August-University Göttingen, Göttingen, Germany; f Department of Geriatrics, Evangelisches Krankenhaus Göttingen-Weende, Göttingen, Germany; g Institute for Microbiology, Centre for Infection Medicine, University of Veterinary Medicine Hannover, Hanover, Germany; h Institute of Immunology, University of Heidelberg, Heidelberg, Germany; i Department of Pathology, University of Veterinary Medicine Hannover, Hannover, Germany

**Keywords:** *Streptococcus suis*, Ide*_Ssuis_*, IgM, opsonization, cysteine protease, IdeS-family protease

## Abstract

*Streptococcus suis* (*S. suis*) causes meningitis, arthritis and endocarditis in piglets. The aim of this study was to characterize the IgM degrading enzyme of *S. suis* (Ide*_Ssuis_*) and to investigate the role of IgM cleavage in evasion of the classical complement pathway and pathogenesis. Targeted mutagenesis of a cysteine in the putative active center of Ide*_Ssuis_* abrogated IgM cleavage completely. In contrast to wt rIde*_Ssuis_*, point mutated rIde*_Ssuis_*_C195S did not reduce complement-mediated hemolysis indicating that complement inhibition by rIde*_Ssuis_* depends on the IgM proteolytic activity. A *S. suis* mutant expressing Ide*_Ssuis_*_C195S did not reduce IgM labeling, whereas the wt and complemented mutant showed less IgM F(ab’)2 and IgM Fc antigen on the surface. IgM cleavage increased survival of *S. suis* in porcine blood *ex vivo* and mediated complement evasion as demonstrated by blood survival and C3 deposition assays including the comparative addition of rIde*_Ssuis_* and rIde*_Ssuis_*_C195S. However, experimental infection of piglets disclosed no significant differences in virulence between *S. suis* wt and isogenic mutants without IgM cleavage activity. This work revealed for the first time *in vivo* labeling of *S. suis* with IgM in the cerebrospinal fluid of piglets with meningitis. In conclusion, this study classifies Ide*_Ssuis_* as a cysteine protease and emphasizes the role of IgM cleavage for bacterial survival in porcine blood and complement evasion though IgM cleavage is not crucial for the pathogenesis of serotype 2 meningitis.

## Introduction

*S. suis* disease is one of the main reasons for high economic losses in pig husbandry worldwide [1]. *S. suis* colonizes the mucosal surfaces of healthy pigs but can lead to invasive disease, mainly in growing piglets of approximately five to ten weeks of age [2,3]. These piglets might develop suppurative meningitis, arthritis, endocarditis, serositis and septicemia upon *S. suis* infection [1]. *S. suis* is also an emerging zoonotic agent especially in Southeast Asia [4].

The species *S. suis* is very heterogeneous. It has been divided into 35 serotypes and more than 700 sequence types (ST) [5]. Serotype 2 is worldwide the most frequently isolated from diseased pigs and humans [4]. However, other serotypes such as serotypes 9, 7, 3 and 1/2 can cause invasive disease as well. The distribution of serotypes depends on the geographical region [4]. However, special adaption to its main host, the pig, is not well understood. A myriad of virulence-associated factors involved in immune evasion have been described for *S suis* [6,7]. Virulence factors involved specifically in complement evasion on the other hand are scarce. Proven or potential candidates are the polysaccharide capsule [8], factor H binding proteins FhB and Fhbp [9] and lastly the IgM protease Ide*_Ssuis_* [10]. Ide*_Ssuis_*, expressed universally among *S. suis* serotypes, is a highly specific protease, with porcine IgM as its sole substrate [11]. Ide*_Ssuis_* shows homology to other streptococcal immunoglobulin degrading enzymes such as IdeS of *S. pyogenes*, IdeZ of *S. equi subsp. zooepidemicus*, IdeE of *S. equi subsp. equi* and IdeP of *S. phocae subsp. phocae* [12]. Ide*_Ssuis_*, however, remains the only specific IgM protease and, in contrast to the above mentioned IgG proteases, possesses a long C-terminal domain not involved in protease activity [11].

IgM has important effector functions in immunity. First of all, IgM represents not only the main class of natural antibodies [13], but is also the first antibody being produced in response to antigenic stimulation [14]. Secondly, IgM is the main activator of the classical complement cascade. It is an estimated 1000 times more potent in activating complement than IgG [15]. Activation of complement leads to opsonophagocytosis, direct killing of bacteria via the terminal membrane attack complex (MAC), induction of inflammation and recruitment of immune cells [16]. Recently, the complement system, traditionally regarded as part of the innate immune system, has been linked to adaptive immunity, especially T-and B-lymphocytes [16]. Both T-and B-lymphocytes possess the IgM receptor FcμR [15] and IgM can enhance the induction of memory B cells in mice [17]. Lastly, monomeric IgM is the only B-cell receptor (BCR) of naïve porcine B-cells since pigs do not express IgD as a BCR [18]. The possession of an IgM protease can be pivotal for pathogens early in infection and in young animals when IgM titers are highest. Previous studies of our group showed, that expression of Ide*_Ssuis_* reduced IgM-mediated C3 deposition on the bacterial surface and has a positive effect on survival of *S. suis* in porcine blood in the presence of specific IgM titers [10]. The aim of this study was to investigate if Ide*_Ssuis_* is a cysteine protease and to specifically test if the IgM cleavage activity of Ide*_Ssuis_* is involved in complement evasion and virulence.

## Results

### Point mutation of cysteine 195 to serine in the putative active center of rIde_Ssuis_ leads to abrogation of IgM cleavage activity

**Figure 1.**
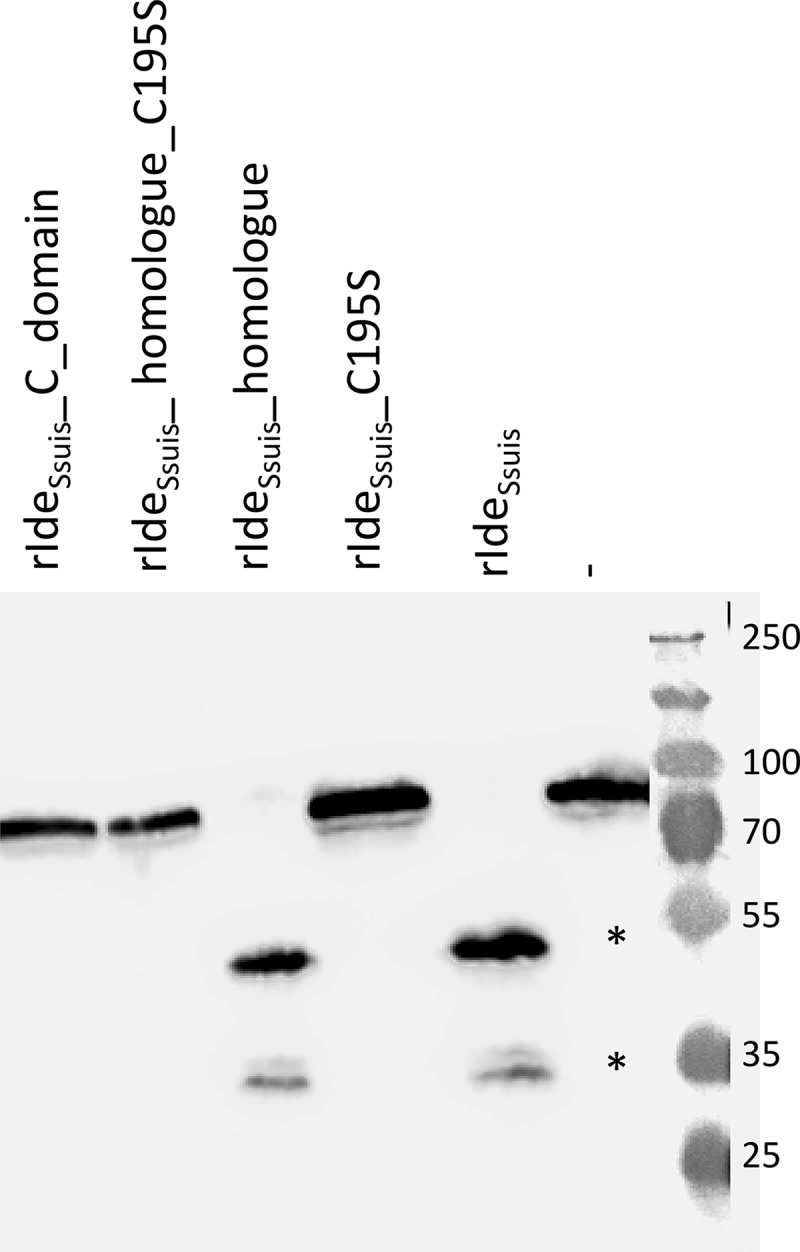
Point mutation of the cysteine 195 within the putative catalytic center of rIde*_Ssuis_* leads to loss of IgM cleavage activity. Porcine serum was incubated with 5 µg/ml of the indicated rIde*_Ssuis_* constructs, followed by anti-pig IgM Western blot analysis with a polyclonal anti-IgM antibody. Serum incubated with phosphate buffered saline served as negative control (-). A 10% percent polyacrylamide gel was used for SDS-PAGE under reducing conditions. Marker bands in kDa are shown on the right-hand side. IgM cleavage products are indicated by asterisks.

Sequence homologies to known streptococcal cysteine proteases and a protease inhibitor profile [11] suggested that the IgM protease Ide*_Ssuis_* is a cysteine protease. In order to proof this hypothesis and further elucidate the role of IgM cleavage by Ide*_Ssuis_* in complement evasion and virulence, a point mutation was introduced into the putative active center of rIde*_Ssuis_*. The cysteine at position 195 was replaced by a serine, the rest of the gene remaining intact. The point mutation was also introduced into the truncated construct rIde*_Ssuis_*_homologue lacking the large C-terminal domain. Expression and purification of full length protein was verified for the different recombinant Ide*_Ssuis_* constructs by Coomassie staining (Fig. S1). Analyzing the IgM cleaving activity of cysteine to serine point mutated recombinant rIde*_Ssuis_*_C195S and rIde*_Ssuis_*_homologue_C195S by Western Blot analysis revealed that both recombinant constructs are unable to cleave porcine IgM (Figure 1). Hence, the presence of a cysteine at position 195 is crucial for IgM cleavage. This finding indicates that Ide*_Ssuis_* is indeed a cysteine protease, in accordance with its homology to the IdeS-family of streptococcal immunoglobulin degrading enzymes.

### Reduction of complement-mediated hemolysis depends on IgM cleavage

**Figure 2.**
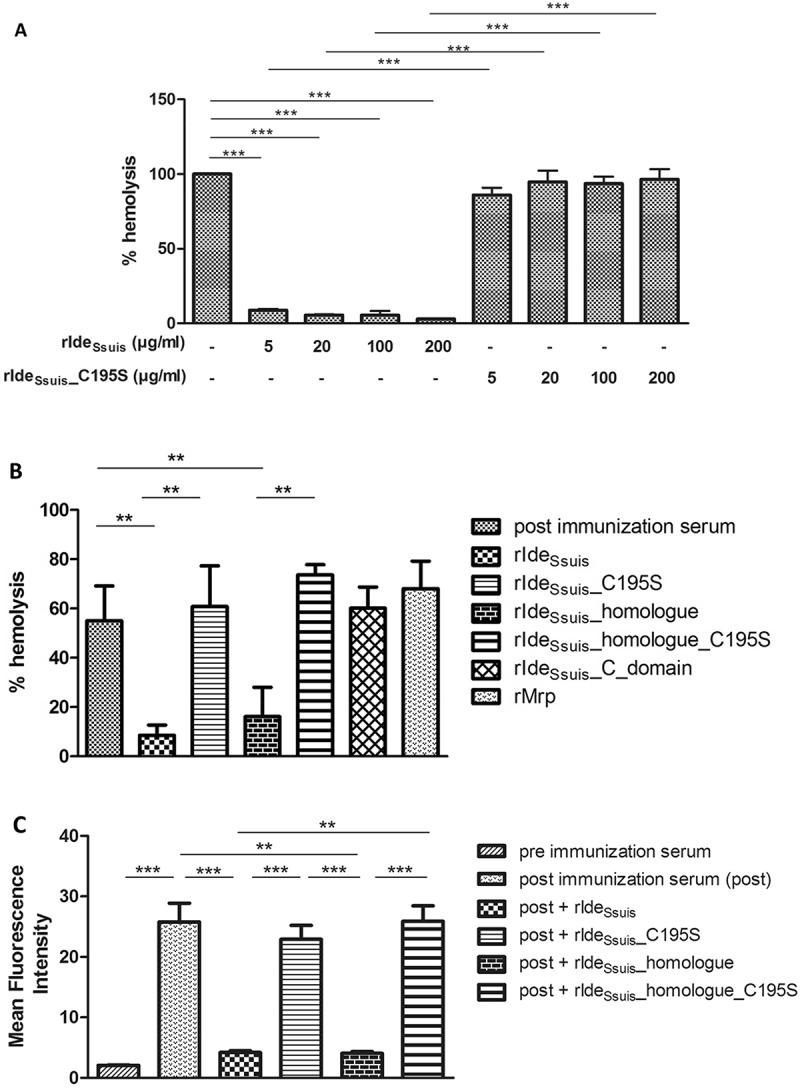
In the presence of high *S. suis* specific IgM titers, complement-mediated hemolysis and labeling of sheep erythrocytes with IgM are significantly reduced by rIde*_Ssuis_* constructs with IgM cleaving activity, but not by rIde*_Ssuis_* constructs lacking IgM cleavage activity due to the C195S point mutation. Hemolysis assays were performed by addition of purified sheep erythrocytes to pig anti-sheep erythrocyte serum which had been pretreated with either different concentrations of rIde*_Ssuis_* and rIde*_Ssuis_*_C195S (a) or different recombinant Ide*_Ssuis_* constructs (b). The assays were performed with porcine serum drawn seven days after immunization with sheep erythrocytes (n = 4). Bars and error bars represent mean and standard deviation and significant differences are indicated. (a) Hemolysis induced by water was defined as one hundred percent and is represented by the first bar. (b) rIde*_Ssuis_* wt, rIde*_Ssuis_*_C195S, rIde*_Ssuis_*_homologue, rIde*_Ssuis_*_homologue_C195S, rIde*_Ssuis_*_C_domain, rMrp were compared regarding their ability to reduce complement mediated hemolysis at 18 µg/ml. Recombinant Mrp served as a control protein and was purified the same way as rIde*_Ssuis_* constructs. Post immunization serum without either rIde*_Ssuis_* construct served as negative control. (c) Flow cytometric analysis of IgM labeled sheep erythrocytes was performed after addition of purified sheep erythrocytes to a heat inactivated porcine anti-sheep erythrocyte serum drawn seven days after immunization and pretreated with 18 µg/ml of the indicated rIde*_Ssuis_* constructs. Serum drawn prior to immunization with sheep erythrocytes served as negative control. Serum drawn seven days post immunization served as positive control. Bars and error bars show mean values and standard deviations (n = 6). Significant differences are indicated by asterisks. Probabilities were considered as follows p < 0.05 *, p < 0.01 **, p < 0.001 ***.

To investigate the role of IgM cleavage in inhibition of the classical complement pathway, a hemolysis assay was conducted. It could be shown that 5 µg/ml rIde_Ssuis_ suffices to reduce complement-mediated hemolysis by over 90% in the presence of high anti-erythrocyte IgM titers (Figure 2(a)) as elicited by prime vaccination of a pig with sheep erythrocytes. Hemolysis could not be reduced in the presence of high anti-erythrocyte IgG titers as shown by hemolysis assays conducted with a porcine anti-erythrocyte serum drawn fourteen days after prime booster immunization (Fig. S2). Hemolysis caused by the classical complement pathway was abrogated only by rIde*_Ssuis_* and rIde*_Ssuis_*_homologue, not by rIde_Ssuis__C195S, rIde_Ssuis__homologue_C195S or rIde*_Ssuis_*_C_domain (Figure 2(b)). Thus, the IgM cleaving activity of rIde*_Ssuis_* alone must be responsible for abrogation of complement activation in the presence of high IgM titers. An impact of the C-terminal domain of Ide*_Ssuis_* on complement activity could not be demonstrated since hemolysis levels remained unaffected by addition of rIde*_Ssuis_*_C-domain (Figure 2(b)). Hemolysis assays in the presence of high anti-erythrocyte IgG titers revealed that none of the tested rIde*_Ssuis_* constructs, even those with IgM protease activity, were able to reduce hemolysis (Fig. S2). In concordance with the results obtained by the hemolysis assay, IgM labeling of sheep erythrocytes was significantly reduced by rIde*_Ssuis_* and rIde*_Ssuis_*_homologue, but not by the point mutated constructs rIde_Ssuis__C195S and rIde*_Ssuis_*_homologue_C195S as shown by flow cytometry (Figure 2(c)).

### Addition of rIde_Ssuis_ and rIde_Ssuis__homologue, but not rIde_Ssuis__C195S and rIde_Ssuis__homologue_C195S promotes survival of S. suis 10∆ide_Ssuis_ in porcine blood ex vivo

**Figure 3.**
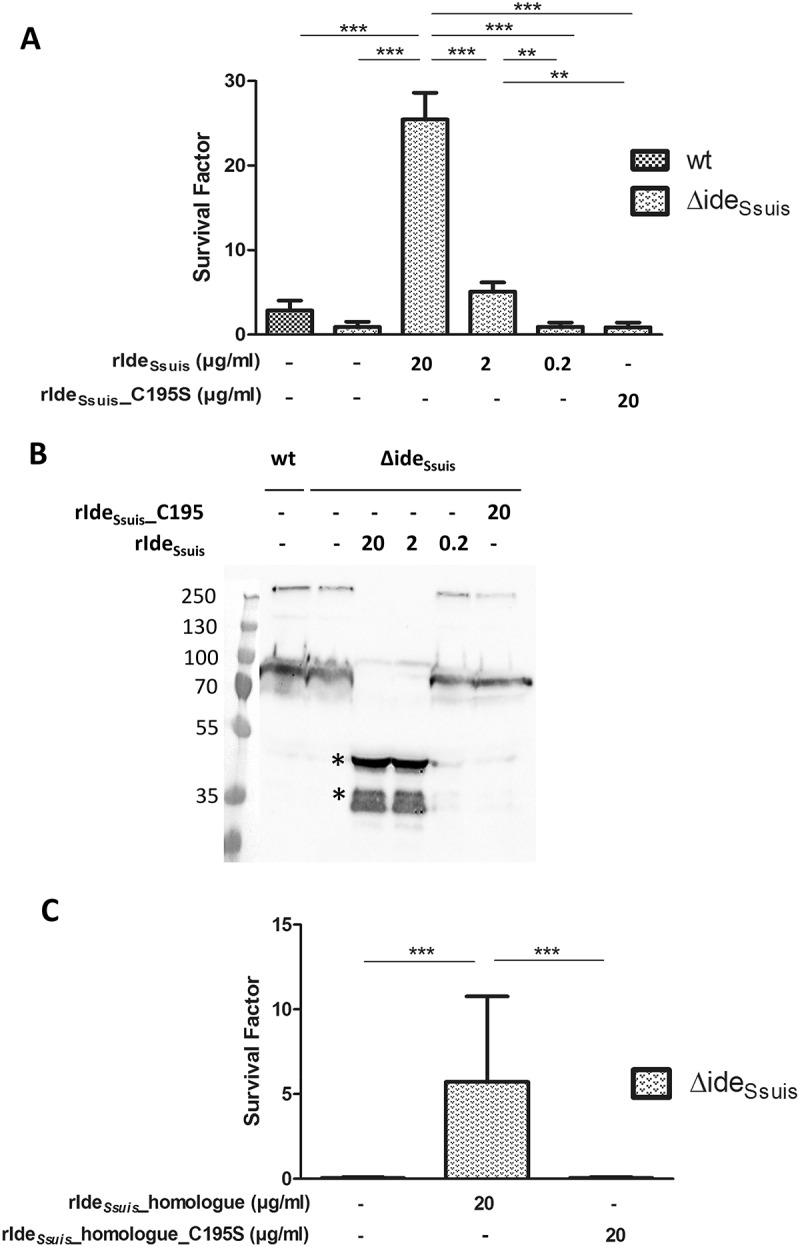
IgM proteolysis by rIde*_Ssuis_* and rIde*_Ssuis_*_homologue promotes survival of *S. suis* 10∆ide*_Ssuis_* in porcine blood. (a) Blood survival assay in whole porcine blood of growing piglets with high specific anti-*S. suis* IgM antibody titers. *S. suis* 10∆ide*_Ssuis_* was supplemented with 0.2, 2, 20 µg rIde*_Ssuis_* or 20 µg rIde*_Ssuis_*_C195S (n = 8) per ml blood. The survival factor of *S. suis* strain 10 and the isogenic deletion mutant 10∆ide*_Ssuis_* in porcine blood with the addition of rIde*_Ssuis_* and rIde*_Ssuis_*_C195S after a two-hour incubation period at 37°C is depicted. Bars and error bars represent means and standard deviations. Significant differences are indicated. Probabilities were considered as follows p < 0.05 *, p < 0.01 **, p < 0.001 ***. (b) Anti-IgM Western blot analysis of plasma after the blood survival assay shown in Figure 4(a). IgM cleavage products were detectable in plasma after a blood survival assay including 2 and 20 µg rIde*_Ssuis_* and as faint bands in association with incomplete cleavage also for 0.2 µg rIde*_Ssuis_*. Marker bands in kDa are shown on the left-hand side. Asterisks indicate IgM cleavage products for the first positive lane. (c) Blood survival assay of *S. suis* 10∆ide*_Ssuis_* in whole porcine blood of growing piglets (n = 6). *S. suis* 10∆ide*_Ssuis_* was supplemented with 20 µg rIde*_Ssuis_*_homologue or rIde*_Ssuis_*_homologue_C195S per ml blood (n = 6). Survival factors were calculated and are depicted analogously to Fig. (A).

Previous work showed that the deletion mutant 10∆ide*_Ssuis_* is attenuated in survival in porcine blood with high specific anti-*S. suis* IgM titers *ex vivo* [10]. To investigate if the attenuation is due to the inability of the mutant to cleave porcine IgM, blood survival assays with external complementation of the mutant with functional rIde*_Ssuis_* and non-functional rIde*_Ssuis_*_C195S were performed. In these assays, *S. suis* 10∆ide*_Ssuis_* could survive significantly better in porcine blood of growing piglets if rIde*_Ssuis_* was externally added (Figure 3(a)). Survival was increased in a concentration depend manner starting at a rIde*_Ssuis_* concentration of 2 µg/ml. Addition of 2 µg/ml rIde*_Ssuis_* led to a survival factor of the deletion mutant that was almost twice as high as that of the wt. Addition of 20 µg/ml rIde*_Ssuis_* increased the survival factor of the deletion mutant to levels almost nine times as high as the survival factor of the wt. Importantly, there was no increase in bacterial survival by addition of rIde*_Ssuis_*_C195S, even at a concentration of 20 µg/ml (Figure 3(a)). In the plasma of samples where rIde*_Ssuis_* had been added to 10∆ide*_Ssuis_*, IgM cleavage products were detectable by anti-IgM Western blot analysis (Figure 3(b)). Cleavage products started to appear at 2 µg rIde*_Ssuis_*/ml. IgM cleavage products were not detectable for blood samples incubated with *S. suis* wt or 10∆ide*_Ssuis_* supplemented with rIde*_Ssuis_*_C195S. A further independent blood survival assay was conducted with the addition of 20 µg/ml rIde*_Ssuis_*_homologue and rIde*_Ssuis_*_homologue_C195S to *S. suis* 10∆ide*_Ssuis_*. This assay confirmed that survival of 10∆ide*_Ssuis_* can only be increased by addition of rIde*_Ssuis_* constructs with IgM cleavage activity (Figure 3(c)). In conclusion, survival of *S. suis* 10∆ide*_Ssuis_* in porcine blood is restricted by IgM under the chosen experimental conditions. Furthermore, IgM cleavage through addition of rIde*_Ssuis_* or rIde*_Ssuis_*_homologue is responsible for the significant increase in survival of the *S. suis* 10∆ide*_Ssuis_* mutant.

**Figure 4.**
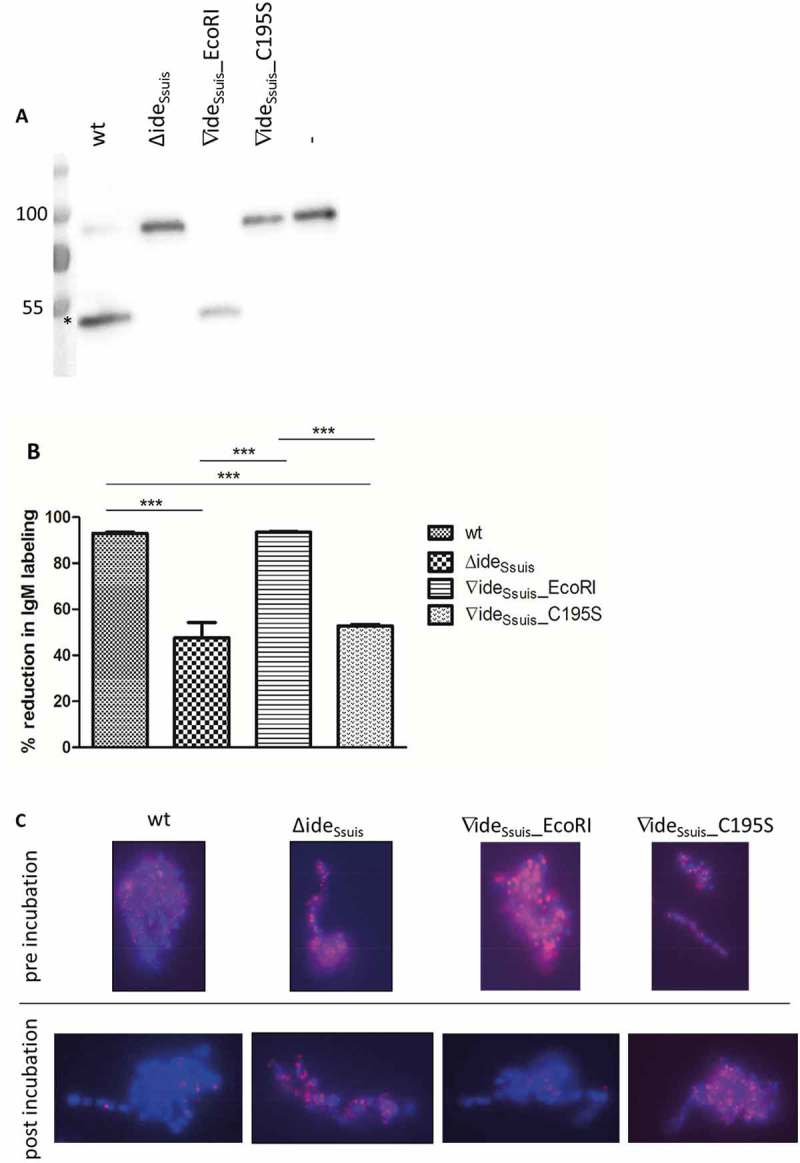
*S. suis* strain 10 (wt) and the complemented mutant 10∆ide*_Ssuis_*∇ide*_Ssuis_*_EcoRI (∇ide*_Ssuis_*_EcoRI) cleave porcine IgM (a) and reduce bacterial IgM labeling by over 90% (B,C), in contrast to 10∆ide*_Ssuis_* (∆ide*_Ssuis_*) and the point mutated mutant 10∆ide*_Ssui_*_s_∇ide*_Ssuis_*_C195S (∇ide*_Ssuis_*_C195S). (a) Twenty-four-fold concentrated supernatants of the respective strains were incubated with 1:100 diluted porcine serum, followed by anti-porcine IgM Western Blot analysis under reducing conditions. Incubation of serum with phosphate-buffered saline was used as negative control (-). An 8% separating gel was used for gel electrophoresis and a polyclonal anti-IgM antibody for detection of IgM. Marker bands in kDa are shown on the left-hand side. Asterisks indicate IgM cleavage products on the left side of the first positive lane. (b, c) The indicated *S. suis* strains were grown to an OD_600_ of 0.8, incubated in anti-*S. suis* serotype 2 hyperimmune serum for 0.5 hours at 4°C and then for four hours at 37°C. IgM labeling of the bacterial surface was analyzed before and after incubation at 37°C by flow cytometry (n = 6) (b) and fluorescent microscopy (c) using a monoclonal anti-IgM antibody and a phycoerythrin (PE)-labeled secondary antibody. The % reduction in IgM labeling was calculated by subtracting the percental amount of IgM positive bacteria after a four-hour incubation period at 37°C from an initially one hundred percent positive population before incubation at 37°C. (c) DAPI (4ʹ,6 diamidino-2-phenylindole) dye in blue was used to stain DNA. IgM, labeled by the monoclonal anti IgM antibody and the PE-labeled secondary antibody appears in pink. Bars and error bars indicate mean and standard deviation. Significant differences are indicated by asterisks. Probabilities were considered as follows p < 0.05 *, p < 0.01 **, p < 0.001 ***.

### Reduction of bacterial labeling with IgM depends on the IgM cleaving activity of Ide_Ssuis_

Two complemented *S. suis* mutants were generated to investigate the role of IgM cleavage by Ide*_Ssuis_* in the pathogenesis of *S. suis* diseases further. Firstly, 10∆ide*_Ssuis_* was chromosomally complemented to render 10∆ide*_Ssuis_*∇ide*_Ssuis_***_** EcoRI expressing wt Ide*_Ssuis_*. A silent mutation (EcoRI site) was introduced in this strain for further discrimination of wt and complemented mutant. Secondly, the cysteine to serine point mutation of Ide*_Ssuis_* was implemented in the chromosome of *S. suis* itself to render ∆ide*_Ssuis_*∇ide*_Ssuis_*_C195S. Site-directed mutagenesis was confirmed using sequencing and Southern blot analysis including confirmation of the silent mutation leading to the new EcoRI site in 10∆ide*_Ssuis_*∇ide*_Ssuis_***_** EcoRI (Fig. S4 and S5). Anti-IgM Western blot analysis showed that the complemented strain 10∆ide*_Ssuis_*∇ide*_Ssuis_*_EcoRI possesses full IgM cleavage activity whereas 10∆ide*_Ssuis_*∇ide*_Ssuis_*_C195S is unable to cleave porcine IgM (Figure 4(a)) even though it expresses a stable Ide*_Ssuis_* antigen (Fig. S3).

**Figure 5.**
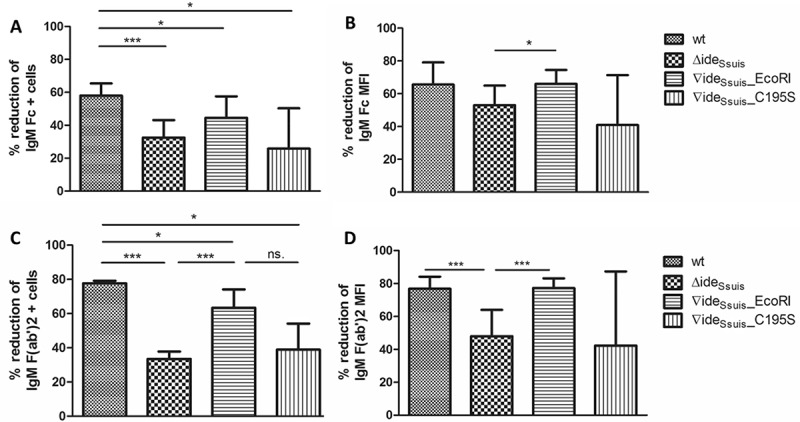
IgM cleavage by Ide*_Ssuis_* reduces surface bound F(ab‘)2 and Fc antigen of porcine IgM. *S. suis* strain 10 (wt), 10∆ide*_Ssuis_* (∆ide*_Ssuis_*), 10∆ide*_Ssuis_*∇ide*_Ssuis_*_EcoRI (∇ide*_Ssuis_*_EcoRI), 10∆ide*_Ssuis_*∇ide*_Ssuis_*_C195S (∇ide*_Ssuis_*_C195S) were incubated in a porcine anti-*S. suis* serotype 2 hyperimmune serum for 0.5 hours at 4°C and then for four hours at 37°C. Bacteria were stained with IgM F(ab‘)2 and IgM Fc specific antibodies and measured by flow cytometry (n = 7) before and after incubation at 37°C. (a) Reduction of the percentage of IgM Fc positive bacteria. (b) Reduction of the geometric mean fluorescence intensity (MFI) of the IgM Fc signal. (c) Reduction of the percentage of IgM F(ab‘)2 positive bacteria. (d) Reduction of the geometric mean fluorescence intensity (MFI) of the IgM F(ab‘)2 signal. The reduction in IgM labeling was calculated by subtracting the percental amount or MFI of IgM positive bacteria after a four-hour incubation period at 37°C from an initially one hundred percent positive population before incubation at 37°C. Bars and error bars indicate mean and standard deviation. Asterisks indicate significant differences. Probabilities were considered as follows p < 0.05 *, p < 0.01 **, p < 0.001 ***.

Opsonization induced by antibodies binding to the bacterial surface is an important immune defense mechanism. Previous work showed that the expression of Ide*_Ssuis_* leads to reduction of surface bound IgM [11]. We hypothesized that point mutation of the cysteine 195 and consequently loss of IgM cleavage activity would lead to the inability of Ide*_Ssuis_* to reduce the amount of surface-bound IgM. *S. suis* wt, 10∆ide*_Ssuis_* and the complemented mutants 10∆ide*_Ssuis_*∇ide*_Ssuis_*_EcoRI and 10∆ide*_Ssuis_*∇ide*_Ssuis_*_C195S were therefore analyzed regarding their ability to reduce labeling of bacteria with IgM. Flow cytometry (Figure 4(b)) and immunofluorescence microscopy (Figure 4(c)) showed that *S. suis* wt and 10∆ide*_Ssuis_*∇ide_Ssuis__EcoRI reduced surface-bound IgM by over 90% in contrast to 10∆ide*_Ssuis_* and 10∆ide*_Ssuis_*∇ide*_Ssuis_*_C195S. Reduction by the wt and complemented mutant was significantly higher than that by the mutants 10∆ide*_Ssuis_* and 10∆ide*_Ssuis_*∇ide*_Ssuis_*_C195S. None of the latter possessed IgM cleaving activity. We hypothesized that upon IgM cleavage, Fc fragments would become detached from the bacterial surface, whereas F(ab’)2 fragments might remain attached to it as Ide*_Ssuis_* cleaves the heavy chain of IgM at the N-terminus of the C3 domain [9]. We therefore analyzed bacterial labeling with IgM using IgM-F(ab’)2 and IgM-Fc specific antibodies, that were tested in Western Blot analysis to recognize the respective parts of porcine IgM only (Fig. S6). Flow cytometry with these specific antibodies revealed that both the percentage of F(ab’)2 positive and Fc positive cells within the *S. suis* population were reduced by Ide*_Ssuis_* activity (Figure 5(a,c)). Analogously, the geometric mean fluorescence intensity (MFI) of both the F(ab’)2 and the Fc signal was reduced by Ide*_Ssuis_* activity (Figure 5(b,d)). The reduction of the IgM-F(ab’)2 signal was generally greater than that of the IgM Fc signal. This was true also for the percentage of the F(ab’)2 and Fc positive cells. Hence it can be concluded that Ide*_Ssuis_*-mediated IgM cleavage leads not only to detachment of IgM Fc fragments form the bacterial surface but of IgM F(ab’)2 fragments as well.

### IgM cleavage by rIde_Ssuis_ reduces deposition of the complement component C3 on the bacterial surface

**Figure 6.**
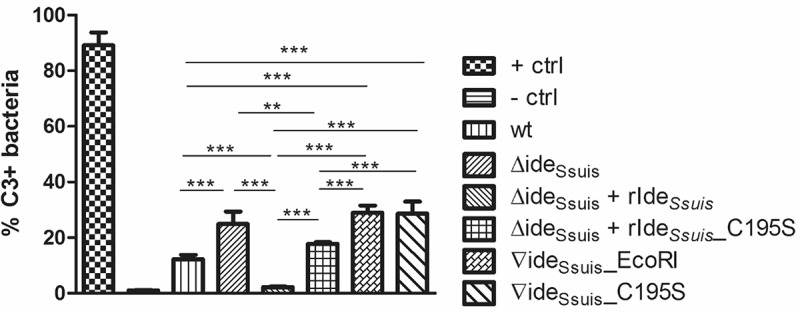
IgM cleavage activity by rIde*_Ssuis_* leads to reduction of C3 antigen on the bacterial surface. *S. suis* strain 10 (wt), 10∆ide*_Ssuis_* (∆ide*_Ssuis_*), 10∆ide*_Ssuis_*∇ide_Ssuis__EcoRI (∇ide*_Ssuis_*_EcoRI), 10∆ide*_Ssuis_*∇ide*_Ssuis_*_C195S (∇ide*_Ssuis_*_C195S) were incubated in a 1:2 diluted IgG depleted porcine anti-*S. suis* serotype 2 hyperimmune serum. After incubation at 37°C, bacteria were stained with a FITC-labeled rabbit anti-human C3c antibody and measured by flow cytometry. *S. suis* strain 10 incubated in undiluted serum served as positive control (+ ctrl). *S. suis* strain 10 incubated with heat-inactivated serum served as negative control (- ctrl). Bars and error bars represent mean and standard deviation. Significant differences are indicated by asterisks. Probabilities were considered as follows p < 0.05 *, p < 0.01 **, p < 0.001 ***.

Differences in surface-bound IgM in the investigated *S. suis* strains led us to hypothesize that the loss of IgM cleavage activity of expressed Ide*_Ssuis_* leads to increased activation of the classical complement pathway and thus increased labeling of bacteria with C3b antigen. Using a serum depleted of IgG antibodies, it was shown that 10∆ide*_Ssuis_* and 10∆ide*_Ssuis_*∇ide*_Ssuis_*_C195S were indeed significantly more labeled with C3 than *S. suis* wt (Figure 6). Surprisingly, also the complemented mutant 10∆ide*_Ssuis_*∇ide*_Ssuis_*_EcoRI was significantly more labeled with C3 than the wt (∇ide*_Ssuis_*_EcoRI: 28.94% C3+ cells; SD: 2.6 vs. wt: 12.18% C3+ cells; SD: 1.6). When rIde*_Ssuis_* was added to 10∆ide*_Ssuis_*, C3 labeling was reduced by 91% (Figure 6). A significant reduction in the percentage of C3 positive cells also occurred when rIde*_Ssuis_*_C195S was added to the deletion mutant, though with 28.5% it was much less pronounced. In fact, 10∆ide*_Ssuis_* supplemented with rIde*_Ssuis_*_C195S was significantly more C3 labeled than 10∆ide*_Ssuis_* supplemented with functional rIde*_Ssuis_* (17.76% C3+ cells; SD: 0.6 vs. 2.22% C3+ cells, SD: 0.3). Furthermore, addition of rIde*_Ssuis_* to 10∆ide*_Ssuis_* led to a significant reduction in C3 labeling even when compared to the level of C3 labeling of the wt (wt: 12.18% C3+ cells; SD: 1.6 vs. 10∆ide*_Ssuis_* + rIde*_Ssuis_*: 2.22% C3+ cells; SD: 0.3). Thence it can be concluded that activation of the classical complement pathway and subsequent C3 labeling of *S. suis* is reduced by the IgM cleaving activity of rIde*_Ssuis_*.

### S. suis survival in porcine blood is restricted by active complement

**Figure 7.**
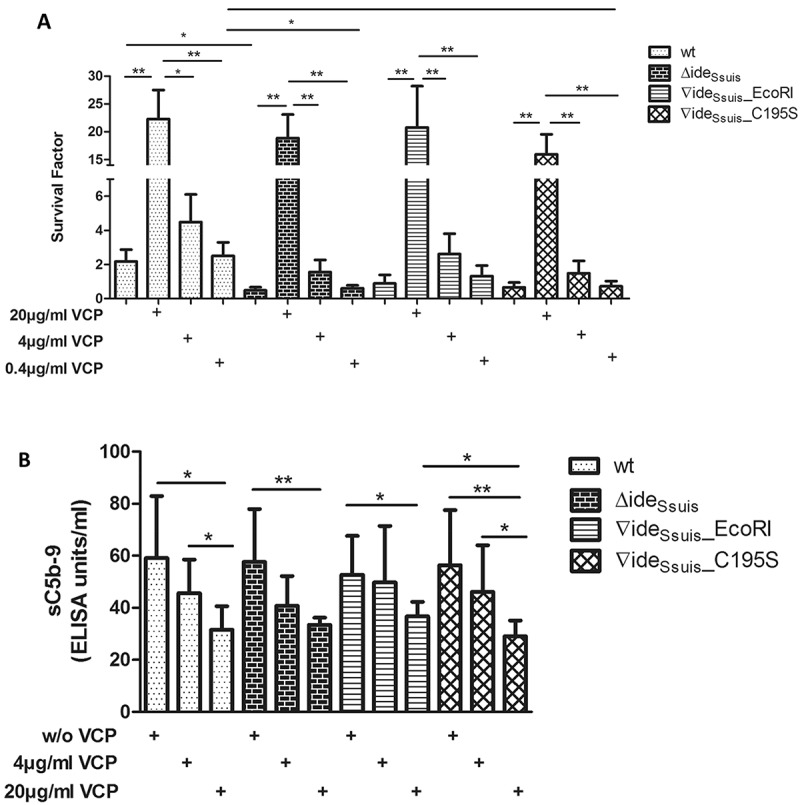
Survival of *S. suis* serotype 2 in porcine blood is restricted by active complement. (a) Survival of *S. suis* strain 10 (wt), 10∆ide*_Ssuis_* (∆ide*_Ssuis_*) and the two complemented strains 10∆ide*_Ssuis_*∇ide*_Ssuis_*_EcoRI (∇ide*_Ssuis_*_EcoRI) and 10∆ide*_Ssuis_*∇ide*_Ssuis_*_C195S (∇ide*_Ssuis_*_C195S) was analyzed in a whole blood survival assay with the addition of the complement inhibitor vaccinia virus complement control protein (VCP). Porcine whole blood from growing piglets (n = 6) with high anti-*S. suis* IgM titers was pre-incubated with 0.4, 4, 20 µg VCP/ml for five minutes at 37°C. 4 × 10^6^ CFU/ml of the indicated *S. suis* strains were then added and the survival factor determined by plate counting after two hours at 37°C. (b) Soluble C5b-9 was determined in porcine plasma after the blood survival assay by sC5b-9 ELISA. A concentration dependent reduction in sC5b-9 levels was detectable after addition of VCP to porcine whole blood (Pearson r values: wt: −0,9487; 10∆ide*_Ssuis_*: −0,8485; 10∆ide*_Ssuis_*∇ide*_Ssuis_*_EcoRI: −0,9999; 10∆ide*_Ssuis_*∇ide*_Ssuis_*_C195S: 0,9821). Bars and error bars indicate mean and standard deviation. Significant differences are indicated by asterisks. Probabilities were considered as follows p < 0.05 *, p < 0.01 **, p < 0.001 ***.

The ability to survive in blood is an important feature for any pathogen causing bacteremia and systemic diseases after dissemination in the blood. We hypothesized that loss of IgM cleavage would lead to increased killing of *S. suis* in porcine blood of growing piglets with high titers of *S. suis* specific IgM. Blood survival assays revealed significantly higher survival factors (SF) for *S. suis* wt than for 10∆ide*_Ssuis_* in the blood of eight-week-old piglets with high anti-*S. suis* IgM (71.4 ELISA units) and high anti-*S. suis* IgG (77.6 ELISA units) antibody titers (Figure 7(a)). *S. suis* wt exhibited also higher survival factors in porcine blood than 10∆ide*_Ssuis_*∇ide*_Ssuis_*_C195S and 10∆ide*_Ssuis_*∇ide*_Ssuis_*_EcoRI, although the differences were not significant (Figure 7(a)). Nevertheless, *S. suis* mutants without IgM cleavage activity (10∆ide*_Ssuis_* and 10∆ide*_Ssuis_*∇ide*_Ssuis_*_C195S) had lower survival factors than *S. suis* wt and 10∆ide*_Ssuis_*∇ide*_Ssuis_*_EcoRI (wt SF: 2.2, SD: 1.7; 10∆ide*_Ssuis_*∇ide*_Ssuis_*_C195S SF: 0.7, SD: 0.7; 10∆ide*_Ssuis_*∇ide*_Ssuis_*_EcoRI SF: 0.9; SD: 1.2; 10∆ide*_Ssuis_* SF: 0.5, SD: 0.48).

To investigate the role of complement in survival of *S. suis* and mutants in porcine blood, the C3 convertase inhibitor vaccinia virus complement control protein (VCP) was added to the blood prior to the addition of bacteria. VCP inhibits both the classical and the alternative C3 convertase and has been shown to inhibit the complement cascade in porcine blood [19]. VCP was used at concentrations where it is known to inhibit complement significantly but not completely [19]. The addition of VCP led to a concentration dependent increase of survival of all strains in porcine blood (Figure 7(a)). Only at concentrations of 20 µg VCP/ml blood the survival of *S. suis* increased significantly above levels without complement inhibitor. Addition of 4 µg/ml VCP led to a doubling of the survival factor of the wt, a triplication of the survival factor of 10∆ide*_Ssuis_* and 10∆ide*_Ssuis_*∇ide*_Ssuis_*_EcoRI and a 2.4-fold increase of the survival factor of 10∆ide_Ssuis_∇ide_Ssuis__C195S, but differences to the samples without VCP were not significant. Furthermore, at 4 µg/ml VCP the survival factors of *S. suis* wt and 10∆ide*_Ssuis_*∇ide*_Ssuis_*_EcoRI were higher than those of 10∆ide*_Ssuis_* and 10∆ide*_Ssuis_*∇ide*_Ssuis_*_C195S although differences were not significant (wt vs. 10∆ide*_Ssuis_* p = 0.109, wt vs. 10∆ide*_Ssuis_* ∇ide*_Ssuis_*_ C195S p = 0.101, 10∆ide*_Ssuis_* ∇ide*_Ssuis_*_EcoRI vs. 10∆ide*_Ssuis_* p = 0.943, 10∆ide*_Ssuis_* ∇ide*_Ssuis_*_EcoRI vs. 10∆ide*_Ssuis_* ∇ide*_Ssuis_*_C195S p = 0.798). Interestingly, addition of 4 µg/ml VCP to 10∆ide*_Ssuis_* led to an increased survival of the mutant approximating *S. suis* wt levels (SF wt: 2.2, SD: 1.712; SF 10∆ide*_Ssuis_* + 4 µg VCP: 1.6, SD: 1.749). As a read-out parameter for complement activation in this assay, soluble C5b-9 (sC5b-9) was measured after the described blood survival assay. The sC5b-9 ELISA revealed a concentration dependent decrease in sC5b-9 levels in samples with VCP (Figure 7(b)). Significant differences in sC5b-9 levels between *S. suis* strains were not detected. In conclusion, survival of *S. suis* in porcine blood is restricted by active complement.

### S. suis wt strain 10 shows higher survival factors than the mutant strains 10∆ide_Ssuis_ and 10∆ide_Ssuis_∇ide_Ssuis__C195S in opsonophagocytosis assays

As tissue infected with *S. suis* is mainly infiltrated by neutrophils, opsonophagocytosis assays were conducted to specifically investigate the role of IgM cleavage in evasion of killing by these immune cells. Opsonophagocytosis assays did indeed reveal that the survival factor of *S. suis* strain 10 (SF mean: 3.06, SD: 1.36) was significantly higher than that of 10∆ide*_Ssuis_* (SF mean: 1.27, SD: 0.85) and 10∆ide*_Ssuis_* ∇ide*_Ssuis_* C195S (mean: 1.36, SD: 0.59) (Fig. S7). However, the survival factor of *S. suis* wt was also significantly higher than that of 10∆ide*_Ssuis_* ∇ide*_Ssuis__*EcoRI (SF mean: 1.6, SD: 0.81).

### The complemented S. suis 10∆ide_Ssuis_∇ide_Ssuis__EcoRI mutant expresses a thinner and less dense polysaccharide capsule

**Figure 8.**
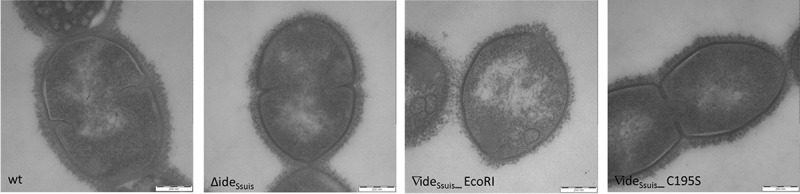
Transmission electron microscopy reveals a different capsule morphology for *S. suis* 10∆ide*_Ssuis_*∇ide*_Ssuis_*_ EcoRI. The expression and thickness of the capsule of *S. suis* strain 10 (wt), 10∆ide*_Ssuis_* (∆ide*_Ssuis_*), 10∆ide*_Ssuis_*∇ide*_Ssuis_*_C195S (∇ide*_Ssuis_*_C195S) and ∆ide*_Ssuis_*∇ide*_Ssuis_*_EcoRI (∇ide*_Ssuis_*_EcoRI) was investigated by transmission electron microscopy using lysine-ruthenium red staining. The morphology of the capsule of the complemented *S. suis* mutant ∇ide*_Ssuis_*_EcoRI differs from that of the other strains in that it appears less dense and significantly thinner.

Blood survival, opsonophagocytosis and C3 deposition assays revealed phenotypic differences between the complemented mutant 10∆ide*_Ssuis_*∇ide*_Ssuis_*_EcoRI and the wt. Despite full IgM cleavage activity, the complemented mutant showed lower survival factors in blood survival and opsonophagocytosis assays and was significantly more C3 labeled than the wt. We conducted comparative transmission electron microscopy of *S. suis* strain 10, 10∆ide*_Ssuis_*, 10∆ide*_Ssuis_*∇ide*_Ssuis_*_EcoRI and 10∆ide*_Ssuis_*∇ide*_Ssuis_*_C195S to verify intact capsule expression in these strains. However, electron microscopy indicated that the morphology of the capsule of 10∆ide*_Ssuis_*∇ide*_Ssuis_*_EcoRI differed from that of the three other investigated strains (Figure 8). The capsule of this complemented strain was less dense and less compact. Additionally, measurements of the capsule thickness revealed that the capsule of 10∆ide*_Ssuis_*∇ide*_Ssuis_*_EcoRI was significantly thinner than that of the wt (mean capsule thickness of 10∆ide*_Ssuis_*∇ide*_Ssuis_*_EcoRI: 40.2 nm, SD: 4.0 vs. mean. capsule thickness of the wt: 60.7 nm, SD: 5.3). Differences in capsule expression between *S. suis* strain 10, 10∆ide*_Ssuis_* and 10∆ide*_Ssuis_*∇ide*_Ssuis_*_C195S were not recorded. PCR analysis of 16 kb of the *cps*2 locus of *S. suis* strain 10 and 10∆ide*_Ssuis_* ∇ide*_Ssuis_*_EcoRI was conducted to investigate if larger deletions or insertions in *cps* genes were detectable in the complemented mutant. However, the *cps* amplification products did not differ in size between *S. suis* strain 10 and 10∆ide*_Ssuis_* ∇ide*_Ssuis_*_EcoRI (Fig. S8). In conclusion, the attenuated phenotype of 10∆ide*_Ssuis_*∇ide*_Ssuis_*_EcoRI might be due to an aberrant capsule morphology, but the reason for this phenotypic alteration is not known.

### IgM cleavage by Ide_Ssuis_ is not crucial for virulence of S. suis serotype 2 in growing piglets

**Figure 9.**
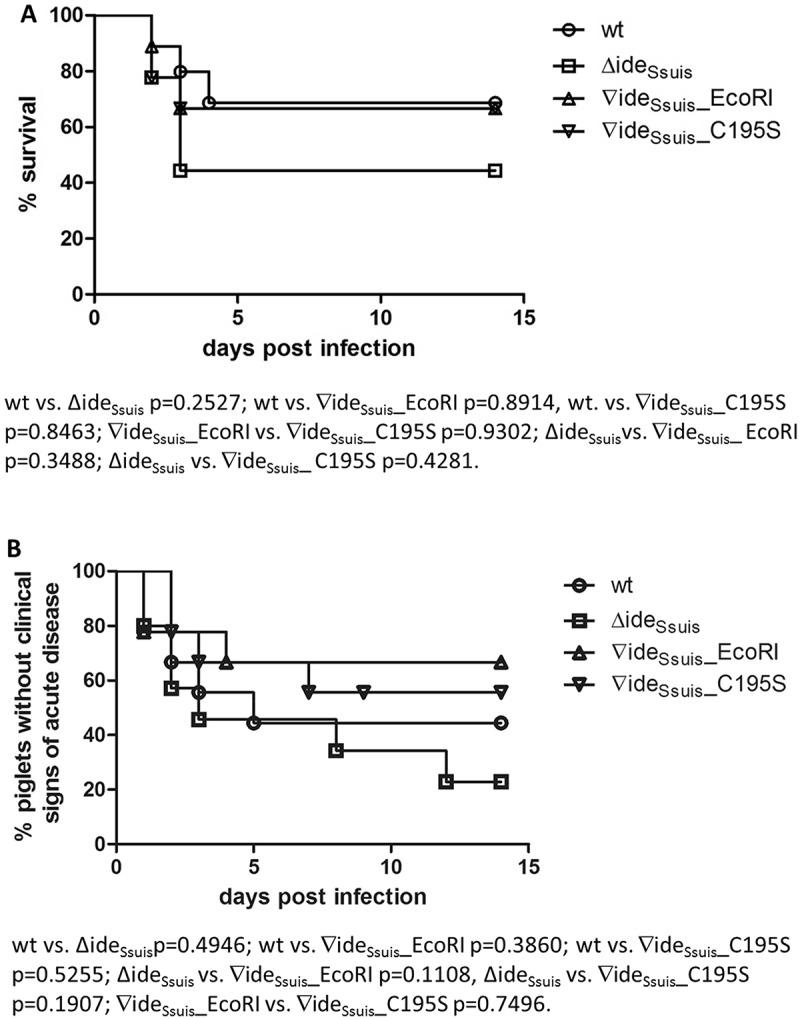
Mortality (a) and morbidity (b) of growing piglets experimentally infected with the indicated *S. suis* strains. Eight-week old piglets were infected with *S. suis* strain 10 (wt), 10∆ide*_Ssuis_* (∆ide*_Ssuis_*), 10∆ide*_Ssuis_*∇ide*_Ssuis_*_EcoRI (∇ide*_Ssuis_*_EcoRI), 10∆ide*_Ssuis_*∇ide*_Ssuis_*_C195S (∇ide*_Ssuis_*_C195S). Morbidity was defined as an inner body temperature equal to or greater than 40.2°C and/or typical clinical signs of *S. suis* disease such as acute lameness or convulsions. Statistical analysis of the Kaplan-Meier diagrams was performed using the log-rank test. P-values are shown below the diagrams.

Former results in our laboratory suggested an attenuation of 10∆ide*_Ssuis_* in prime-vaccinated growing piglets [20]. Data on bacterial survival in porcine blood generated in this study also hinted at a potential attenuation of *S. suis* mutants without IgM cleavage activity. An animal infection experiment was consequently conducted to investigate the role of IgM cleavage by Ide*_Ssuis_* in the pathogenesis of *S. suis* serotype 2 infection by comparing the mutant 10∆ide*_Ssuis_*∇ide*_Ssuis_*_C195S to the wt. Immunological screening of the piglets prior to infection revealed moderate to high anti-*S. suis* IgM (mean of 52.2 ELISA units over all infection groups) and IgG (mean of 129.6 ELISA units over all infection groups) titers and very low anti-Ide*_Ssuis_* antibody titers (mean of 1.5 ELISA units over all infection groups) (Fig S.9). Antibody titers did not differ significantly between the infection groups. Sixty-seven percent of piglets infected with either the wt, 10∆ide*_Ssuis_*∇ide*_Ssuis_*_EcoRI or 10∆ide*_Ssuis_*∇ide*_Ssuis_*_C195S survived the experiment versus 44.4% of piglets infected with 10∆ide*_Ssuis_* (Figure 9(a)). In the wt, 10∆ide*_Ssuis_*, 10∆ide*_Ssuis_*∇ide*_Ssuis_*_C195S and 10∆ide*_Ssuis_*∇ide*_Ssuis_*_EcoRI infection groups four, three, five and six out of nine animals each showed no clinical signs of disease, respectively (Figure 9(b)). Pathological screenings revealed that five of nine 10∆ide*_Ssuis_*, three of nine 10∆ide*_Ssuis_*∇ide*_Ssuis_*_C195S and three of nine wt infected animals had moderate or severe fibrinous-suppurative meningitis (Table 1). Thus, meningitis was found as often or even more often in piglets infected with strains not cleaving IgM. The pathohistological scores for suppurative and fibrinous inflammations of predefined tissues were as follows (ranging from 0 to 5): wt infected group ω = 1.78, 10∆ide*_Ssuis_* infection group ω = 2.78, 10∆ide*_Ssuis_*∇ide*_Ssuis_*_EcoRI group ω = 1.22, 10∆ide*_Ssuis_*∇ide*_Ssuis_*_C195S ω = 1.89 (Table 1). The respective infection strains were re-isolated from both the brain and the cerebrospinal fluid (CSF) of all piglets with signs of central nervous system disorder (Table 2). Additionally, the infection strains could be reisolated from various inner organs, namely spleen, liver, lung, endocard and peritoneum (Table 2). EcoRI restriction enzyme digest of ide*_Ssuis_*-PCR products of reisolates from the wt, the 10∆ide*_Ssuis_*∇ide*_Ssuis_*_C195S and the 10∆ide*_Ssuis_*∇ide*_Ssuis_*_EcoRI infection groups demonstrated that the complemented strain had kept both the ide*_Ssuis_* gene and the EcoRI cleavage site within since it was clearly distinguishable from the wt and 10∆ide*_Ssuis_*∇ide*_Ssuis_*_C195S by a 799 bp EcoRI cleavage product. Sequencing of the ide*_Ssuis_* gene of all reisolates of the 10∆ide*_Ssuis_*∇ide*_Ssuis_*_C195S infection group confirmed that the C195S mutation was still present. In conclusion, IgM cleavage of *S. suis* serotype 2 is not crucial for causing meningitis in growing piglets.

**Table 1.** Histopathological scoring of fibrinosuppurative lesions of growing piglets challenged with the indicated S. *suis* strains.

			brain			serosae			joint				spleen, liver				lung			heart			
			meningitis, chorioiditis			pleuritis, peritonitis			synovialitis				splenitis, hepatitis				pneumonia			endocarditis			
infection strain^a^	piglets w/o lesions	piglets with lesions ≥ 3 locations	5^b^	3^c^	1^d^	4^b^	2^c^	1^d^	4^b^	2^c^	1^d^		4^b^	2^c^	1^d^		4^b^	2^c^	1^d^	4^b^	2^c^	1^d^	ω^e^
wt	4/9	0/9	3/9	0/9	0/9	0/9	0/9	0/9	0/9	0/9	0/9		0/9	0/9	2/9		0/9	0/9	0/9	0/9	0/9	0/9	1.89
∆ideSsuis	4/9	0/9	5/9	0/9	0/9	0/9	0/9	0/9	0/9	0/9	0/9		0/9	0/9	3/9		0/9	1/9	0/9	0/9	0/9	0/9	2.78
∇ideSsuis_EcoRI	6/9	0/9	1/9	2/9	0/9	0/9	0/9	0/9	0/9	0/9	0/9		0/9	0/9	1/9		0/9	0/9	0/9	0/9	0/9	0/9	1.22
∇ideSsuis_C195S	5/9	0/9	3/9	0/9	0/9	0/9	0/9	1/9	0/9	0/9	0/9		0/9	0/9	2/9		0/9	0/9	0/9	0/9	0/9	0/9	1.78

^a^Infection strains were *S. suis* strain 10 (wt), 10∆ide*_Ssuis_* (∆ide*_Ssuis_*), 10∆ide*_Ssuis_*∇ide*_Ssuis_*_EcoRI (∇ide*_Ssuis_*_EcoRI), 10∇ide*_Ssuis_* ∇ide*_Ssuis_*_C195S (∇ide*_Ssuis_*_C195S).

^b^Scores of 4 or 5 were assigned to moderate to severe diffuse or multifocal fibrinosuppurative inflammations

**^c^**Scores of 3 or 2 were assigned to mild focal fibrinosuppurative inflammations

**^d^**A score of 1 was assigned to individual single perivascular neutrophils

^e^ω = ∑score_max_/n_animals_

**Table 2.** Reisolation of the infection strains from piglets infected with the indicated *S.suis* strains.

infection strain^a^	no. of piglets with an isolate of the infection strain in ≥ 1 inner organ^b^	no. of piglets with indicated site of infection strain^a^ isolation/total number of piglets								
		tonsils	lung^c^	serosa^d^	spleen	liver	brain, CSF^e^	joint fluid^f^	endocard	blood
wt	3/9	3/9	0/9	0/9	3/9	2/9	3/9	0/9	0/9	0/9
∆ideSsuis	5/9	3/9	1/9	0/9	5/9	4/9	5/9	0/9	2/9	1/9
∇ideSsuis_EcoRI	3/9	4/9	1/9	1/9	1/9	2/9	3/9	0/9	0/9	1/9
∇ideSsuis_C195S	3/9	3/9	1/9	0/9	3/9	3/9	3/9	0/9	1/9	0/9

^a^Infection strains were *S. suis* strain 10 (wt), 10∆ide*_Ssuis_* (∆ide*_Ssuis_*), 10∆ide*_Ssuis_*∇ide*_Ssuis_*_EcoRI (∇ide*_Ssuis_*_EcoRI), 10∇ide*_Ssuis_* ∇ide*_Ssuis_*_C195S (∇ide*_Ssuis_*_C195S).

^b^Isolates exclusively from the tonsils were not considered

^c^The left cranial lobe was investigated

^d^Pleural, peritoneal or pericardial cavity

^e^Cerebrospinal fluid

^f^Aspirates of both left and right carpal and tarsal joints were investigated in each animal

### Investigation of putative IgM cleavage in vivo

**Figure 10.**
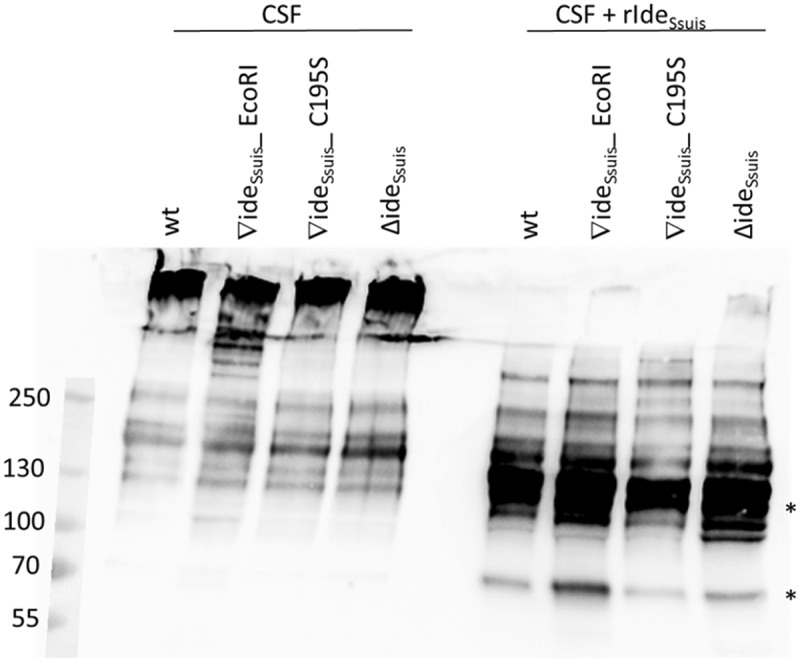
IgM Western blot analysis of cerebrospinal fluid (CSF) of experimentally infected piglets with acute meningitis and high intracerebrospinal *S. suis* burden reveals only intact IgM. Piglets were intranasally infected with the indicated strains and the CSF of one pig with meningitis of each infection group was analyzed for the presence of IgM cleavage products by anti-IgM Western Blot analysis under non-reducing conditions with a monoclonal anti-IgM antibody. Protein concentrations in the CSF were adjusted before loading the 6% polyacrylamide gel. As a control (lanes 7–10), CSF of the same piglets was incubated with 5 µg/ml rIde*_Ssuis_*. Asterisks indicate IgM cleavage products for the last positive lane. Marker bands (in kDa) are shown on the left-hand side.

**Figure 11.**
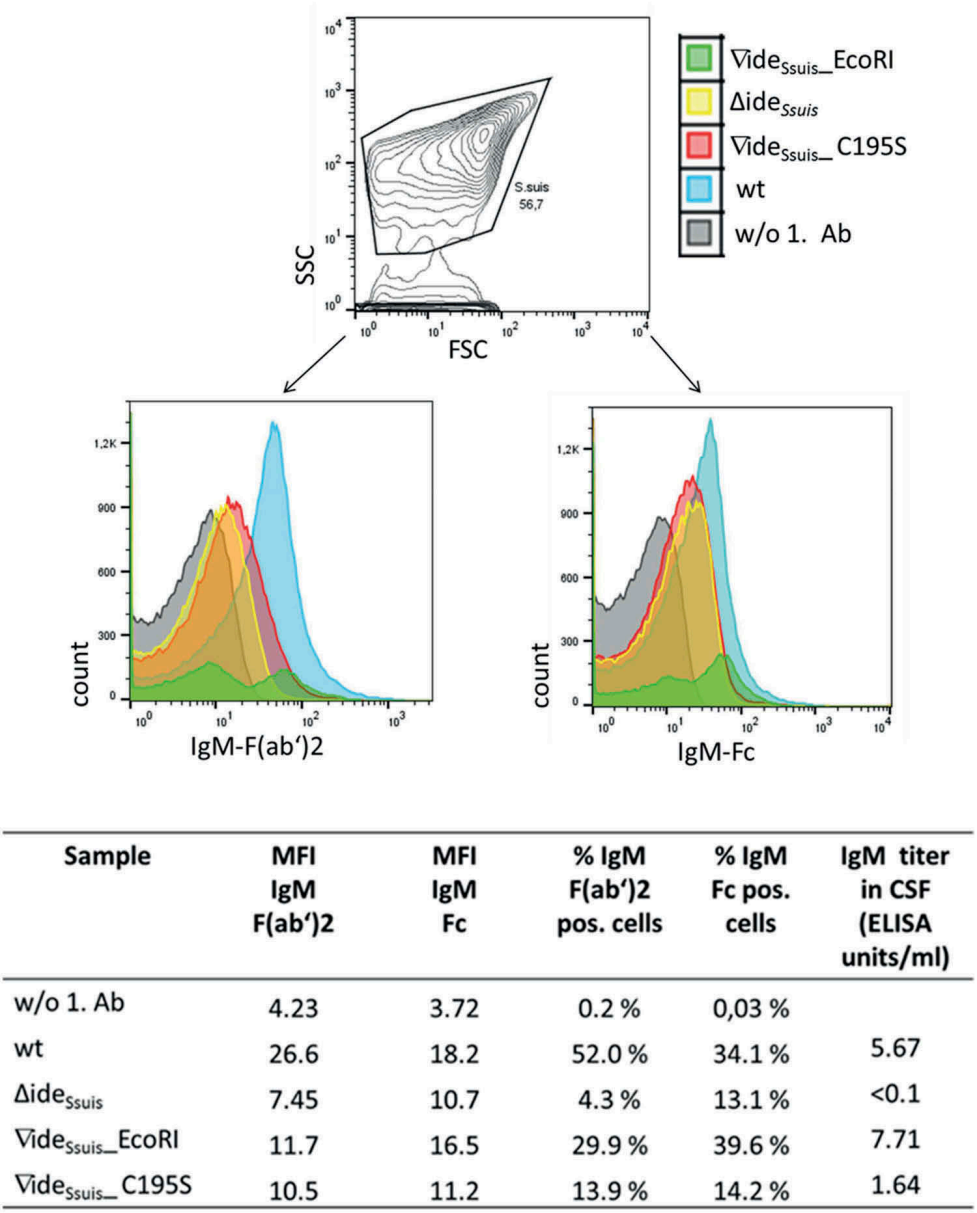
*S. suis* in the CSF of experimentally infected piglets is labeled with porcine IgM. Eight-week old piglets were intranasally infected with *S. suis* strain 10 (wt), 10∆ide*_Ssuis_* (∆ide*_Ssuis_*), 10∆ide*_Ssuis_*∇ide*_Ssuis_*_EcoRI (∇ide*_Ssuis_*_EcoRI) and 10∆ide*_Ssuis_*∇ide*_Ssuis_*_C195S (∇ide*_Ssuis_*_C195S), respectively. The CSF of one pig with meningitis of each infection group was centrifuged and the intracerebrospinal *S. suis* population stained with anti-IgM F(ab’)2 and anti-IgM Fc specific primary antibodies and phycoerythrin (PE) and fluorescein (FITC) labeled secondary antibodies. Bacteria were analyzed for the presence of IgM on their surface via flow cytometry. *S. suis* stained without the first antibodies served as negative control (w/o 1. Ab). The upper panel shows the gating strategy used to define the *S. suis* population. The lower panels shows the *S. suis* population in the CSF of infected piglets as overlay histograms of the F(ab’)2 signals (left hand side) and the Fc signals (right hand side). The table beneath the figure shows the geometric mean fluorescence intensity (MFI), the percentage of IgM F(ab’)2 and IgM Fc positive bacteria and the IgM titers of the four representative piglets.

The CSF of one piglet with meningitis per infection group was investigated for detection of IgM cleavage products by anti-IgM Western Blot analysis. The chosen piglets all had a high intracerebrospinal *S. suis* burden (3–5 x 10^7^ CFU/ml) but varying intracerebrospinal anti-*S. suis* IgM titers (< 0.1–7.7 ELISA units/ml). IgM cleavage products were not detected in the CSF of these experimentally infected piglets with acute meningitis (Figure 10, left). Addition of rIde*_Ssuis_* to the CSF of the same piglets, however, gave rise to IgM cleavage products (Figure 10, right). *S. suis* in the CSF of the same animals was analyzed via flow cytometry for detection of IgM on the bacterial surface using anti IgM F(ab’)2 and anti IgM Fc specific antibodies. *S. suis* in the CSF of experimentally infected piglets was labeled with porcine IgM regardless of the infection strain (Figure 11). However, differences between the individual animals existed regarding the percentage of IgM F(ab’)2 and IgM Fc positive bacteria. In summary, bacteria labeled with uncleaved IgM, as well as bacteria without IgM on the bacterial surface could be detected for all strains. However, IgM labeling seemed to depend on the intracerebrospinal IgM concentration rather than the *S. suis* strain’s ability to cleave IgM. Animals with intracerebrospinal IgM concentrations greater than 5.6 ELISA units/ml had a higher percentage of IgM F(ab’)2 positive and IgM Fc positive cells than those with lower IgM titers. In conclusion, flow cytometric analysis indicated that IgM might still be bound to the bacterial surface of *S. suis* wt in the CSF of piglets with acute meningitis, at least in the case of intracerebrospinal IgM concentrations above five ELISA units.

## Discussion

Previous research indicated, that the IgM protease Ide*_Ssuis_* of *S. suis* is involved in complement evasion [10]. However, as conclusions were based on the comparison of the wt to the mutant 10∆ide*_Ssuis_* only, it remained to be shown that the identified phenotypes depend on the lack of the IgM cleavage activity. Noteworthy, Ide*_Ssuis_* is a very large protein compared to other immunoglobulin proteases and the C-terminal 674 amino acids of the protein are not necessary for cleavage of IgM [11]. This suggests that Ide*_Ssuis_* might carry out an additional function, which might also be related to complement evasion. Thus, we designed experiments to specifically investigate the role of IgM cleavage, which is a unique phenotype of this bacterial pathogen. To this end, a single point mutation (cysteine to serine) was introduced in the putative active center of the IgM cleaving domain of rIde*_Ssuis_* and the isogenic mutant 10∆ide*_Ssuis_* was chromosomally complemented to express wt Ide*_Ssuis_* (10∆ide_Ssuis_∇ide*_Ssuis_*EcoRI), as well as point mutated Ide*_Ssuis_* (10∆ide_Ssuis_∇ide_Ssuis__C195S). As strain 10∆ide_Ssuis_∇ide*_Ssuis_*_C195S expresses stable, point mutated Ide*_Ssuis_* lacking the IgM cleavage activity, this strain is a state-of-art tool to reveal phenotypes determined by this specific protease activity. Comparative investigations with this strain demonstrated that Ide*_Ssuis_-*mediated modulation of IgM labeling is due to the IgM cleaving activity alone. Loss of function experiments and comparative phenotypic analysis adding rIde*_Ssuis_* and rIde*_Ssuis_*_C195S indicated further that Ide*_Ssuis_-*mediated modulation of C3 deposition is also mainly due to the IgM cleavage activity. However, as addition of rIde*_Ssuis_*_C195S also reduced C3 deposition on the bacterial surface significantly, it is not unlikely that the large C-terminus carries out a further function related to complement evasion.

Homology to known cysteine proteases and an inhibitor profile [11] suggested that Ide*_Ssuis_* is a cysteine protease. The inability of cysteine to serine point mutated rIde*_Ssuis_* to cleave porcine IgM, as shown in this study, confirms Ide*_Ssuis_* as a cysteine protease. Thus, Ide*_Ssuis_* is a true member of the IdeS-family of cysteine proteases annotated under the name of C66 in the MEROPS peptidase database (https://www.ebi.ac.uk/merops/cgi-bin/famsum?family=C66).

Since IgM is the most important activator of the classical complement cascade, we investigated the impact of IgM cleavage on the complement system. Mechanisms of complement evasion are multiple in pathogenic bacteria, viruses and yeast. *Streptococcus pyogenes* for example has been shown to use the C5a and C3 peptidase ScpA [21], M protein mediated C4BP binding [22] and the C5b-C9 inhibitory protein SIC [23] to circumvent complement. Gram negative *Borrelia burgdorferi* inhibits complement via multiple FH binding proteins [24] and enterohemorrhagic *Escherichia coli* can cleave C1-INH and thus enhance its complement inhibitory function [25]. For *S. suis* complement evasion has not been intensively studied and so far only the sialic acid containing capsule of certain serotypes and factor H binding molecules have been described as complement evasion factors [26,27]. However, complement plays an important role in immunity to *S. suis* disease. By using the complement inhibitor VCP we show here that survival of *S. suis* in porcine blood is very much restricted by active complement. Accordingly, C3 knockout mice are known to be highly susceptible to disseminated *S. suis* infection [27].

Hemolysis caused by a porcine anti-erythrocyte immune serum with high IgM titers is inhibited by addition of rIde*_Ssuis_*, suggesting that Ide*_Ssuis_* is involved in a novel complement evasion mechanism [11]. In this study, we show that this inhibition depends entirely on the presence of a cysteine at position 195 in the putative active center of Ide*_Ssuis_*. Since C195S point mutated rIde*_Ssuis_* did not influence complement-mediated hemolysis, the IgM cleaving activity of Ide*_Ssuis_* alone must be responsible for reduction of complement mediated hemolysis. In accordance, IgM labeling of sheep erythrocytes was only significantly reduced by functional rIde*_Ssuis_* but not by rIde*_Ssuis_*_C195S.

IgM labeling of *S. suis* can be a prerequisite for subsequent surface-associated complement activation. We therefore analyzed IgM labeling of *S. suis* wt and mutants deficient in IgM cleavage and could show that labeling of *S. suis* with IgM is significantly reduced by Ide*_Ssuis_* activity and that this reduction depends on the presence of a cysteine in the active center of Ide*_Ssuis_*. Interestingly, not only the Fc fragments of porcine IgM detach from the bacterial surface upon Ide*_Ssuis_*-mediated IgM cleavage, but the F(ab’)2 fragments disengage also. This phenomenon contrasts with labeling of bacteria with IgG antibodies upon cleavage by IdeS. Björk et al. showed that IgG Fab fragments remain attached to the bacterial surface upon IgG cleavage [28]. We propose that the detachment of IgM F(ab’)2 fragments is due to the lower affinity of IgM binding [29]. Disruption of the pentameric IgM structure could lead to weaker binding of IgM F(ab’)2 fragments to the bacterial surface and lead to subsequent detachment. If the release of IgM F(ab’)2 and Fc fragments modulates host responses remains to be determined. For IgG Fc fragments a priming effect on neutrophils has been reported [30]. Since neutrophils do not possess Fcµ or Fcα/µ receptors it remains questionable if they are pre-activated by IgM Fc cleavage products also.

Previous experiments with the specific classical complement pathway inhibitor EGTA MgCl_2_ and unencapsulated *S. suis* showed that C3 deposition on *S. suis* depends on the classical complement pathway [10]. The importance of the classical complement pathway in immunity to streptococcal infection was demonstrated by Brown et al. who identified it as the dominant pathway required for innate immunity to *Streptococcus pneumoniae* in mice [31]. We could show in this study, that the reduction of surface bound C3 through Ide*_Ssuis_* expression of *S. suis* wt depends mainly on its IgM cleavage activity. The increased C3 deposition of the complemented mutant 10∆ide_Ssuis_∇ide*_Ssuis_*EcoRI is, to our minds, related to a defective capsule architecture of the mutant which was detected by transmission electron microscopy. Noteworthy, C3 deposition is also significantly enhanced on the surface of an isogenic unencapsulated mutant of *S. suis* serotype 2 strain 10 [27]. We are still confident to conclude that the reduced C3 deposition on the surface of the wt is influenced by IgM cleavage since strains 10∆ide*_Ssuis_* and 10∆ide*_Ssuis_*∇ide*_Ssuis_*_C195S showed a significant increase in C3 labeling in comparison to the wt and 10∆ide*_Ssuis_* showed significantly less C3 deposition after addition of rIde*_Ssuis_* than after addition of rIde*_Ssuis_*_C195S. It was recently pointed out by Kerr et al. that C3 is not only important during systemic disease but also at the site of infection during the initial steps of infection with pneumococci [32]. Additionally, it was shown that resistance to C3b opsonization is important for persistence of pneumococci in the host nasopharynx [31–33] and that complement hinders pneumococci from invasiveness by containing them to mucous membranes [34]. Since *S. suis* asymptomatically colonizes the nasopharynx of pigs, reduced C3b deposition mediated by preceding IgM cleavage could also be important for colonization of the porcine nasopharynx by *S. suis* and eventual spreading. Reduced opsonization with complement nonetheless remains very important for survival in blood which is a prerequisite for *S. suis* to reach sites of infection, especially the cerebrospinal fluid and the meninges. Multiple virulence mechanisms must play in concert to enable *S. suis* to circumvent killing during bacteremia. Both mutants deficient in IgM proteolysis, 10∆ide*_Ssuis_* and 10∆ide*_Ssuis_*∇ide*_Ssuis_*_C195S, are attenuated in whole blood survival assays. Importantly, attenuation of 10∆ide*_Ssuis_* in porcine blood is abolished by addition of rIde*_Ssuis_* but not rIde*_Ssuis_*_C195S. We thus conclude that survival of *S. suis* in porcine blood with specific IgM titers is influenced by the ability to cleave IgM and that survival is very much restricted by IgM. In this study, we could also demonstrate that the two *S. suis* mutants 10∆ide*_Ssuis_* and 10∆ide*_Ssuis_*∇ide_Ssuis__C195S survive less well in opsonophagocytosis assays with purified neutrophils than the wt. This distinguishes Ide*_Ssuis_* from IdeS of *S. pyogenes* because the IgG cleaving activity of IdeS does not impact killing by neutrophils as shown by Okumura et al [35].

Animal experiments conducted by Xiao et al. [36] and our group [10] already indicated that Ide*_Ssuis_* is not an essential virulence factor, despite *ex vivo* data suggesting a role of IgM cleavage by Ide*_Ssuis_* in pathogenicity. Animal experimental infection as conducted in this study confirmed that Ide*_Ssuis_* activity is not crucial for induction of severe pathologies, in particular meningitis, and associated mortality in growing piglets. Analogously, IgM-labeled *S. suis* bacteria were detected in the CSF of piglets from all infection groups. Differences in IgM labeling between the individual animals could possibly be due to varying stages of disease progression and inflammation and thus different amounts of IgM in the CSF at the time of CSF acquisition. Different peak patterns of the fluorescence intensity for IgM-labeled *S. suis* wt and 10∆ide*_Ssuis_*∇ide*_Ssuis_*_EcoRI in the CSF of experimentally infected piglets could not be explained by differing IgM titers or bacterial load within the CSF. Disparate peak patterns might be due to as jet unknown host factors or a divergent behavior of the complemented ide*_Ssuis_* mutant in the CSF under *in vivo* conditions, particularly considering its aberrant capsule morphology. However, none of our conclusions on the impact of IgM cleavage are depended on finding an explanation for the two peaks of the complemented *S. suis* mutant since it expresses stable Ide*_Ssuis_* and is able to cleave IgM. We conclude that Ide*_Ssuis_* activity does not suffice to reduce labeling of *S. suis* with IgM in the CSF, especially in a highly inflammatory setting such as acute meningitis. This would also explain why we could not detect significant differences in the number of animals with meningitis among infection groups. However, to our knowledge, this is the first detection of IgM-labeled streptococci in the CSF of experimentally infected animals. Ide*_Ssuis_* activity might not be enough to reduce labeling of *S. suis* with IgM completely in an inflammatory setting but IgM cleavage could have an important role for *S. suis’* ability to survive in the nasopharynx since IgM concentrations are lower in nasal secretion and saliva than in serum [37] or inflamed tissue. Our findings that Ide*_Ssuis_-*mediated IgM cleavage does not influence virulence significantly, resemble results published for IdeS of *S. pyogenes* which was also shown unessential for virulence of a highly invasive Group A streptococcus strain [35]. Okumura et al. proposed that the reason for this is the highly virulent strain background and that immunoglobulin cleavage might be important for less virulent strains [35]. The same might be true for Ide*_Ssuis_* since all investigations were carried out with a highly virulent serotype 2 strain and derived mutants.

Former studies on Ide*_Ssuis_* related virulence were not specifically designed to reveal the role of IgM proteolysis in pathogenesis and a complemented mutant was not included. The experimental design of this study, including a mutant differing from the wt in one amino acid only, allowed to investigate the role of Ide*_Ssuis_*-mediated IgM cleavage in virulence more thoroughly. Accordingly, we can exclude that the phenotypic comparison is influenced by functions of the large C-terminus of Ide*_Ssuis_*, which are not yet known.

Despite the finding that Ide*_Ssuis_* is not an essential virulence factor, the fact that this protease is conserved among *S. suis* serotypes [11] points to an evolutionary important function. Ide*_Ssuis_* might not be essential for induction of disease in growing piglets but it might play a role in colonization of mucous membranes. It has recently been shown that human secretory IgM, actively secreted to mucous membranes, can activate complement and offer protection at mucosal surfaces [38]. Therefore, cleavage of IgM by Ide*_Ssuis_* might be crucial for *S. suis* to circumvent activation of complement on mucous membranes and thus facilitate colonization. Furthermore, since monomeric IgM is the only virgin B-cell receptor in swine, pigs lacking IgD [39], IgM cleavage might inhibit B-cell activation and thus have far reaching consequences for cell mediated immunity. It has been shown that FcµR, expressed on B-lymphocytes, is an uptake receptor for IgM labeled pathogens [40]. It is therefore reasonable to assume that cleavage of IgM bound to the bacterial surface might hinder uptake of IgM labeled bacteria by B-lymphocytes and thus encumber their antigen presenting ability. Further B-lymphocyte functions such as antibody production and memory cell formation could also potentially be inhibited or at least modulated by Ide*_Ssuis_*-mediated IgM cleavage. Complement inhibition through IgM cleavage could for example reduce C3d mediated B-cell co-stimulation via CD21. Further studies are needed to investigate this question, in particular the importance of Ide*_Ssuis_* in colonization as well as in disease progression and immune modulation.

In summary, in this study, the IgM cleaving activity of Ide*_Ssuis_* of *S. suis* was investigated regarding complement modulation, survival in porcine blood and virulence. Whereas the IgM cleaving activity of Ide*_Ssuis_* was shown to be important for reduction of complement-mediated hemolysis, labeling of sheep erythrocytes with IgM and opsonization of *S. suis* with IgM and complement, an impact of IgM cleavage in induction of disease, especially meningitis, could not be demonstrated.

We conclude, that Ide*_Ssuis_* of *S. suis* is a cysteine protease and its IgM cleaving activity is important for bacterial survival in porcine blood and evasion of the classical complement pathway, but not for virulence in growing piglets. Including numerous *in vitro* assays as well as an *in vivo* model of infection, this study provides extensive insight into the role of IgM cleavage in *S. suis* pathogenesis.

## Materials and methods

### Bacterial strains and growth conditions

*S. suis* strain 10 is a virulent serotype 2 strain that has been used by different groups in studies on pathogenesis [11,41,42]. All other strains used in this study were derived from strain 10 by targeted mutagenesis. The isogenic mutant 10∆ide*_Ssuis_* (∆ide*_Ssuis_*) carries an in-frame deletion of the complete ide*_Ssuis_* gene except the signal sequence [11]. The other mutants, 10∆ide*_Ssuis_*∇ide*_Ssuis_*_EcoRI (∇ide*_Ssuis_*_EcoRI) and 10∆ide*_Ssuis_*∇ide*_Ssuis_*_C195S (∇ide*_Ssuis_*_C195S) were constructed in this study. *S. suis* was grown in Todd-Hewitt broth (THB) (Becton Dickinson, catalogue #249,240) and on Columbia agar plates with 6% sheep blood (Oxoid, catalogue #PB5039A) at 37°C. *Escherichia coli* was grown in Luria-Bertani (LB) medium (Carl Roth, catalogue #X968.2) at 37°C under constant shaking. The following antibiotics were added to the medium if needed: 100 µg/ml ampicillin for *E. coli* carrying a pET45b-derived plasmid, 8 µg/ml and 3.5 µ/ml chloramphenicol for *E. coli* and *S. suis* carrying a pSET5s-derived plasmid, respectively.

### DNA techniques, primers and sequencing

Standard DNA manipulations were performed as described [43]. Chromosomal DNA of *S. suis* strain 10 served as template for PCR. Primers were designed based on the ide*_Ssuis_* sequence of *S. suis* P1/7 (Gene ID: 8153996) which is identical to the ide*_Ssuis_* sequence of strain 10 [11]. Primer sequences are listed in Table S1. PCR was conducted using Phusion polymerase (New England Biolabs, catalogue #M0530L) and sequencing was performed by Seqlab (Göttingen, Germany).

### Targeted mutagenesis of pETide_Ssuis_

The plasmid pETide*_Ssuis_* [11] was point mutated using QuikChange Lightning Multi Site Directed Mutagenesis Kit (Agilent Technologies, catalogue #210513). Plasmid DNA served as template for the mutagenesis primer PrimerC184S. This primer created a single base exchange in ide*_Ssuis_* at the position 195 resulting in the replacement of a cysteine by a serine in the final protein. Following thermal cycling, a DpnI digest was conducted and the product electro transformed into competent *E. coli* BL21. A similar procedure was carried out to generate rIde*_Ssuis_*_homologue_C195S using QuikChange Lightning Site Directed Mutagenesis Kit (Agilent Technologies, catalogue #210518). More precisely, pETide*_Ssuis_*_homologue, a plasmid encoding the His-tagged N-terminal domain of Ide*_Ssuis_* (aa 36 to 432), sufficient for IgM proteolysis [11], was used as template DNA for PCR with the primers IdeSsuis_C195S_for and IdeSsuis_C195S_rev. The final constructs pETide*_Ssuis_*_C195S and pETide*_Ssuis_*_homologue_C195S were sequenced to verify the original sequences and the codon encoding the C195S point mutation.

### Expression and purification of recombinant his-tagged proteins

*E. coli* bacteria harboring the respective plasmids were incubated in LB-broth with ampicillin until an OD_600_ of 0.5. Once the OD was reached, isopropyl-β-D-thiogalactopyranoside (IPTG) (VWR, catalogue #AC121) at a final concentration of 1mM was added and the cultures were then incubated at room temperature (RT) for 2.5h under constant shaking. Following centrifugation, *E. coli* were subjected to a lysozyme digest (1 mg/ml lysozyme 4°C 30 min) followed by ultrasound treatment (Brandson sonifier, 5mm tip, 35% amplitude, 1min 45sec total with 10 sec pulse on, 20sec pulse off). Recombinant proteins were then purified under native conditions via Ni-TED columns as recommended by the manufacturer (Macherey-Nagel, Protino Ni-TED, catalogue #745120.25) and dialyzed against phosphate buffered saline using dialysis membranes with a molecular weight cut off of 6000–8000Da (Carl Roth, ZelluTrans,catalogue #E660.1) and frozen in liquid nitrogen. Recombinant muramidase-released protein (rMrp) was purified as previously described [44]. His-tagged proteins were subjected to SDS-polyacrylamide gel electrophoresis and stained by Coomassie (expedeon, InstantBlue, catalogue #SKU: ISB1L).

### SDS-PAGE and Western blot analysis

SDS-PAGE was performed with 10 or 8% separating gels under reducing conditions or 6% separating gels under non-reducing conditions. Stacking gels were uniformly 4%. Following gel electrophoresis proteins were blotted onto nitrocellulose membranes (Carl Roth, catalogue #HP40.1) and blocked with 5% skim milk powder in Tris-buffered saline with 0.05% Tween 20 (TBST). Incubation with primary and secondary antibodies was performed in TBST with 1% skim milk powder. Antibody dilutions are specified in Table S2. Protein detection was performed using chemiluminescent substrates SuperSignal West Pico (Thermo Fisher, catalogue #34,079) or Quantum (Biozym, catalogue #541013).

### Analysis of IgM cleavage activity of recombinant Ide_Ssuis_

Determination of IgM cleavage was performed as described previously [11]. Briefly 5 µg/ml recombinant protein was added to a 1:100 dilution of porcine serum. The whole was incubated at 37°C for 2.5h on a rotator, followed by anti IgM Western blot analysis under reducing conditions.

### Complement hemolysis assay

Recombinant proteins (rIde*_Ssuis_*, rIde*_Ssui_*_s__C195S, rIde*_Ssuis_*_homologue, rIde*_Ssuis_*_homologue_C195S, rIde*_Ssuis_*_C-domain, rMrp) were incubated in a concentration of 18 µg/ml with a 1:100 dilution of porcine anti-sheep erythrocyte (αEry) serum drawn either 7 or 14 days post immunization with sheep erythrocytes [10] for 1.5 h at 37°C on a rotator in a total volume of 0.25ml as described previously [10]. For investigating complement inhibition in hemolysis assays, rIde*_Ssuis_* and rIde*_Ssuis_*_C195S were added to the sera prior to the hemolysis assay to reach final concentrations of 5, 20, 100, 200 µg/ml. Sheep erythrocytes were purified as described [10] and adjusted to a 2% suspension with sodium chloride. Erythrocytes and pre-incubated sera were then mixed at a ratio of 1:1 (100 µl each) in a 96-well plate (V-bottom) and incubated for 30 min at 37°C on an orbital shaker. Plates were then centrifuged (5 min 1000g RT) and the OD_405_ of the supernatant was measured.

### Detection of IgM and IgG on the surface of ovine erythrocytes

The detection of antibodies on ovine erythrocytes was conducted as described previously [10]. Briefly, αEry serum (both 7d and 14d post immunization) was incubated at 56°C for 30min to inactivate complement. Recombinant proteins were then added (18µg/ml) and the whole incubated for 1.5h at 37°C on a rotator. Subsequently, a 2% sheep erythrocyte suspension was added at a ratio of 1:1 (50 µl each) and the mixture incubated at 4°C for 45min on a rotator. Erythrocytes were then centrifuged, blocked with 5% goat serum for 1h at 4°C and subsequently washed with phosphate buffered saline with a pH of 7.4 (PBS). A mouse anti-pig IgM antibody (Serotec catalogue #MCA637) at a dilution of 1:225 or a goat anti-pig IgG antibody (Serotec, catalogue #AHP865P) at a dilution of 1:10,000 was then added and the samples incubated for 1h at 4°C. Cells were washed twice and incubated for 1h at 4°C in the dark with the following secondary antibodies: phycoerythrin (PE)-labeled goat anti-mouse IgG (BioLegend, catalogue #405307) at a dilution of 1:500 for detection of porcine IgM and Alexa Fluor 488-labeled chicken anti-goat IgG (Life Technologies, catalogue #A-21467) at a dilution of 1:800 for detection of porcine IgG. Samples were then washed twice and fixated with 0.375% formaldehyde and measured by flow cytometry (BD FACSCalibur).

### Complementation and targeted mutagenesis of S. suis 10∆ide_Ssuis_

*S. suis* 10∆ide*_Ssuis_* was chromosomally complemented using the thermosensitive suicide vector pSET5s [45] to render the two mutants *S. suis* 10∆ide*_Ssuis_*∇ide*_Ssuis_*_C195S and 10∆ide*_Ssuis_*∇ide*_Ssuis_*_EcoRI. The former mutant contains a point mutation in the ide*_Ssuis_* gene leading to a serine instead of a cysteine at the position 195 of the final protein. The later mutant contains a silent mutation at the position 1793 of ide*_Ssuis_* resulting in a new EcoRI cleavage site enabling distinction of the complemented strain from the wt. Chromosomal DNA of *S. suis* strain 10 served as template in a PCR with the primers preProIdeSsuisPstI, postEndIdeSsuisBamHI to introduce the cleavage sites for the restriction enzymes BamHI and PstI. The PCR product, as well as the pSET5s vector were then digested with the two indicated enzymes and afterwards ligated to render pSET5side*_Ssuis_*. To introduce the C195S encoding mutation in ide*_Ssuis_*, pSET5side*_Ssuis_* plasmid DNA was used in a PCR with the mutagenesis primers IdeSsuis_C195S_for, IdeSsuis_C195S_rev resulting in pSET5side*_Ssuis_*_C195S. To introduce the EcoRI cleavage site, primers EcoRI1793ideSsf, EcoRI1793ideSsr were used to create pSET5side*_Ssuis_*_EcoRI. PCR products were DpnI digested and transformed into competent *E. coli* CC118. Sequencing of the whole ide*_Ssuis_* reading frame confirmed the presence of both point mutations at the correct sites. The plasmids pSET5side*_Ssuis_*_C195S and pSET5side*_Ssuis_*_EcoRI were then transformed into competent *S. suis* 10∆ide*_Ssuis_* (25µF, 2.5kV 200Ω, Genpulser, Biorad). Temperature shifts, performed as described previously [46], ensured allelic exchange and later excision of the vector from *S. suis*.

### Genotypic analysis of S. suis mutants

To verify the correct chromosomal complementation, mutants were sequenced, tested in restriction enzyme digest with EcoRI and analyzed by non-radioactive Southern blot. For Southern blotting, chromosomal DNA was digested with HincII, blotted onto a positively charged nylon membrane (Biorad, catalogue #162–0165) and subsequently hybridized with two different biotinylated probes. Between hybridizations with the different probes, the membrane was stripped with 0.2 N NaOH, 1% sodium dodecyl sulfate (SDS). The first probe, 450 bp in size, binding a 685bp fragment within ide*_Ssuis_* was constructed using the primers Sonde450bpfor and Sonde450bprev. The second probe, 412bp in size, binding within the pSET5s backbone was generated with the primer pair pSET5sSondefor and pSET5sSonderev. After hybridization, the membrane was incubated with peroxidase-labeled streptavidin (Biolegend, catalogue #405210) and signals detected by chemiluminescence (blots see Fig. S5). PCR analysis with the primers pSET5spreMCSfor and postMCSpSET5s was conducted to verify complete excision of the pSET5s plasmid from the *S. suis* chromosome.

### Phenotypic analysis of S. suis mutants for Ide_Ssuis_ expression and IgM cleavage

Expression of Ide*_Ssuis_* was confirmed in bacterial supernatants by anti-Ide*_Ssuis_* Western blot analysis as described [11]. Furthermore, the mutants’ ability to cleave porcine IgM was tested by incubation of concentrated culture supernatants with porcine serum, followed by anti-IgM Western blotting. Concentration of supernatants was performed as described [11]. Briefly, 50 ml cultures of *S. suis* strain 10, 10∆ide*_Ssuis_*, 10∆ide*_Ssuis_* ∇ide*_Ssuis__*EcoRI, ∆ide*_Ssuis_*∇ide*_Ssuis_*_C195S were grown to an OD_600_ of 0.8, centrifuged and the supernatants concentrated 24-fold using 30 kDa cut off centrifugal filters (Merck Milipore, catalogue #UFC903024). One hundred microliter of the 24-fold concentrated supernatants was incubated with a 1:100 dilution of porcine serum, followed by anti-IgM Western blotting.

### Transmission electron microscopy

The expression and thickness of the capsule of *S. suis* strain 10, 10∆ide*_Ssuis_*, 10∆ide*_Ssuis_*∇ide*_Ssuis_*_C195S and 10∆ide*_Ssuis_*∇ide*_Ssuis_*_EcoRI was investigated by transmission electron microscopy using lysine-ruthenium red (LRR) staining as described previously [47]. The capsule thickness of *S. suis* strain 10 and 10∆ide*_Ssuis_*∇ide*_Ssuis_*_EcoRI was measured 25 times. Measurements were only conducted of bacteria which capsule had been cut in a 90° angle.

### Generation of porcine IgM (Fab’)2 and IgM Fc specific antibodies

To generate an antibody specifically against porcine IgM F(ab’)2 fragments, porcine IgM was purified from serum, digested with rIde*_Ssuis_* and F(ab’)2 fragments, purified as described below, were used as antigen for immunization of a rabbit. IgM-Fc specific antibodies were purified from a commercial polyclonal anti-pig IgM antibody (biomol, catalogue #A100-117A). All chromatographic purification procedures were performed with the ÄCTA prime plus chromatography system (GE Healthcare, Little Chalfont, United Kingdom). In detail, the gamma globulin fraction of porcine serum was twice precipitated with 40% ammonium sulfate, followed by dialysis against phosphate buffered saline with 300 kDa cut off membranes (Spectra/Por Float A-Lyzer G2, Spectrum Labs, catalogue #G235072) for five days. The dialysate was then affinity chromatographed over protein G columns (HiTrap protein G column, GE Healthcare via VWR, catalogue #29–0485-81) to exclude porcine IgG. The flow through from protein G columns was purified by thiophilic adsorption chromatography (HiTrap IgM purification columns, GE Healthcare via VWR, catalogue #17–5110-01). The eluate from thiophilic adsorption chromatography was purified further via size exclusion chromatography (HiLoad 16/600 Superdex 200pg, GE Healthcare via VWR, catalogue #28–9893-35). The eluates corresponding in size to pentameric IgM were pooled and concentrated via 10 kDa cut off centrifugal filters (Sartorius, catalogue #VS2001) to render a total volume of 2 ml. Purified IgM was next subjected to a digest with rIde*_Ssuis_* (20 µg rIde*_Ssuis_*/mg IgM) for 2.5h at 37°C. F(ab’)2 fragments were purified from the digest via size exclusion chromatography. The eluates corresponding to 130kDa F(ab’)2 fragments were pooled and subsequently purified via affinity chromatography with a Ni-sepharose column (HisTrap HP, GE Healthcare via VWR, catalogue #29–0510-21) to exclude remaining rIde*_Ssuis_* that had been used to digest IgM. The flow through of this chromatography was then used as antigen for immunization of a rabbit (BioGenes GmbH). The rabbit was immunized three times with 250–500 µg antigen being used for priming and 100 µg antigen for the two booster immunizations. Final bleeding serum was precipitated with 40% ammonium sulfate, the precipitate was then purified by affinity chromatography with Sepharose columns covalently coupled with porcine IgG to exclude those antibodies cross reacting with the light chain of porcine IgG. The flow through was purified further by affinity chromatography with Sepharose columns covalently coupled with porcine IgM-F(ab’)2 fragments generated also by rIde*_Ssuis_* digest. The eluate from this chromatography was analyzed for its specificity for porcine IgM-F(ab’)2 portion in Western blot analysis as described above (Fig. S6). Western blot analysis confirmed that the antibody does not cross-react with Ide*_Ssuis_* and rMrp (results not shown). For purification of anti-IgM Fc specific antibodies, a commercial polyclonal goat anti-pig IgM antibody was purified by affinity chromatography with Sepharose columns coupled with IgM-F(ab’)2. The flow through from this procedure was then applied to Sepharose columns coupled with intact pentameric porcine IgM. The eluate from this chromatography was also verified in Western blot analysis to recognize only IgM Fc fragments (Fig. S6).

### Generation of a porcine hyperimmune serum against S. suis serotype 2 and depletion of IgG from this serum

A pig was prime-booster-booster vaccinated with a *S. suis* bacterin based on strain 10, essentially as described previously for a bacterin vaccination trial [48] except that two booster immunizations were conducted. The generation of hyperimmune sera in piglets is approved by the Landesdirektion Sachsen (permit no. N01/16). This porcine hyperimmune serum was depleted from all IgG using affinity chromatography. The hyperimmune serum was purified via a Protein G sepharose column (GE Healthcare via VWR, catalogue #17–0405-01) using the chromatography system ÄCTA prime plus (GE Healthcare, Little Chalfont, United Kingdom). To assure that no IgG was remaining in the flow through, it was send over the Protein G column again. The IgG free flow through was concentrated using 10 kDa centrifugal filters (Sartorius, catalogue #VS2001).

### Detection of IgM on the surface of S. suis

For detection of IgM on the bacterial surface *S. suis* strain 10, 10∆ide*_Ssuis_*, 10∆ide*_Ssuis_* ∇ide*_Ssuis_*_EcoRI and ∆ide*_Ssuis_*∇ide*_Ssuis_*_C195S were grown to an OD_600_ of 0.8, followed by incubation at 4°C with the anti-*S. suis* hyperimmune serum against *S. suis* serotype 2 for 0.5 h. Hereafter, bacteria were incubated in THB at 37°C for 4h. In the first staining protocol, bacteria were labeled with a mouse anti-pig IgM primary antibody (Serotec, catalogue #MCA 637) and a PE-labeled goat anti-mouse IgG secondary antibody (BioLegend, catalogue #405307) as described by Seele et al. [11]. Presence of IgM was then detected on the surface of *S. suis* using flow cytometry (BD, Fortessa) and immunofluorescent microscopy. The percent reduction of IgM labeling was calculated as follows: The amount of IgM labeled bacteria after opsonization with serum and before incubation at 37°C was set at 100%. The reduction in IgM labeling was then calculated by subtracting the % of IgM positive bacteria after incubation at 37°C from the initial 100%. Staining was also performed with antibodies directed specifically against the F(ab’)2- and Fc-regions of porcine IgM. Prior to this staining, opsonized bacteria were blocked with 1:100 diluted donkey serum (Dianova, catalogue # 017–000-121) at 4°C for 0.5h. Staining with primary and secondary antibodies was performed for 0.5 h at 4°C each. Rabbit anti-pig IgM-F(ab’)2 and goat anti-pig IgM-Fc primary antibodies were both used at a dilution of 1:100. As secondary antibodies, 1:250 dilutedPE-labeled F(ab’)2 donkey anti-goat IgG (Thermo Fisher Scientific, catalogue #31860) and 1:200 diluted fluorescein isothiocyanate (FITC)- labeled donkey anti-rabbit IgG (Dianova, catalogue #DAB-87179) antibodies were used. Detection of IgM labeled *S. suis* was again performed by flow cytometry (BD FACSCalibur). The % reduction in the IgM F(ab’)2 and IgM Fc signal was calculated as described above. To detect IgM on the surface of *S. suis* in the CSF of experimentally infected piglets, CSF of piglets with high intracerebrospinal *S. suis* burden was centrifuged and stained with anti-IgM F(ab’)2 and anti IgM Fc specific antibodies. Cerebrospinal fluid was frozen within five minutes after acquisition and stored at −80°C until analysis. Flow cytometry (BD, FACSCalibur) was used to detect IgM F(ab’)2 and IgM Fc labeling on *S. suis*. To gate *S. suis* in the CSF correctly, preliminary tests were conducted with *S. suis* wt incubated in the CSF of healthy but *S. suis* infected piglets or in the CSF of the same piglets supplemented with serum with moderate IgM levels (50 ELISA units) or IgG levels (29.1 ELISA units), respectively. The frequencies of parent of IgM F(ab’)2 and IgM Fc-positive *S. suis* were determined via FlowJo^TM^_V10 software.

### C3 deposition on the surface of S. suis

Complement deposition on the streptococcal surface was assessed basically as described [10], with the differences of the use of an IgG depleted porcine anti-*S. suis* strain 10 serum as source of IgM and complement. Briefly, *S. suis* strain 10, 10∆ide*_Ssuis_*, 10∆ide*_Ssuis_* ∇ide*_Ssuis_*_EcoRI, ∆ide*_Ssuis_*∇ide*_Ssuis_*_C195S were grown to an OD_600_ of 0.8. Then, 75µl of the respective cultures were centrifuged and incubated for 1h at 37°C on a rotator with 150 µl of a 1:2 dilution of an anti*-S. suis* strain 10 hyperimmune serum depleted of all IgG. Staining of C3 labeled bacteria was conducted with 250µl of a 1:150 diluted FITC-labeled cross-reactive rabbit anti-human C3c antibody (Dako, catalogue #F020102-2) for 1h at 4°C under constant rotation. As positive control, undiluted serum was used, as negative control, serum that had been heat inactivated at 56°C for 30 min was used. For samples with the addition of recombinant protein, 5µg of rIde*_Ssuis_* or rIde*_Ssuis_*_C195S were added to 10∆ide*_Ssuis_* prior to incubation with serum. Samples were measured using BD, FACSFortessa and analyzed using FlowJo^TM^_V10 software.

### Detection of anti-S. suis IgM, anti-S. suis IgG and anti-Ide_Ssuis_ antibodies

Anti*-S. suis* strain 10 IgM, anti*-S. suis* strain 10 IgG and anti-Ide*_Ssuis_* antibody titers were determined by ELISA as described by Seele et al. [20]. Antibody titers below 10 ELISA units were considered as low, ten to seventy ELISA units were considered as moderate and ELISA units greater than 70 were considered as high.

### Blood survival assays

Survival of *S. suis* wt and mutants in porcine blood was analyzed using whole heparinized blood from eight-week old piglets from a commercial pig farm (infected with various *S. suis* serotypes). The withdrawal of blood was approved under the permit number N19/14 by the responsible authorities of the state of Saxony, Germany (Landesdirektion Sachsen). Assays with the addition of rIde*_Ssuis_*_homologue and rIde*_Ssuis_*_homologue_C195S were conducted with blood from eleven-week old piglets known to be free of *S. suis sly*^+^ *mrp*^+^ *epf*^+^ *cps2, cps*7 and *cps*9 that served as placebo animals in an infection experiment approved by the responsible authorities of the state of Saxony, Germany under the permit number TVV 37–17. Survival factors were determined by dividing the number of CFUs after a 2 h incubation period at 37°C by the number of CFUs at t = 0 (CFU were determined by plating of serial dilutions). As indicated, 0.5 ml porcine whole blood was mixed with 0.1, 1, 10 µg rIde*_Ssuis_* or 10 µg rIde*_Ssuis_*_C195S, rIde*_Ssuis_*_homologue, rIde*_Ssuis_*_homologue_C195S. Then 1.5 x 10^6^ CFU *S. suis* wt or 10∆ide*_Ssuis_* were added. Bacteria were added from glycerol stocks prepared at the late exponential growth phase. The mixture was incubated for 2 h at 37°C on a rotator. For the detection of IgM cleavage products after the 2 h incubation period, samples were centrifuged and the supernatant analyzed by anti-pig IgM Western blot analysis under reducing conditions as described above.

For survival assays with the complement inhibitor *vaccinia virus complement control protein* (VCP, GeneBalance, Inc., catalogue #GB-VCP250), 10, 2, 0.2 µg of VCP were added to 0.5 ml whole porcine blood and incubated at 37°C for 5 min on a rotator. *S. suis* wt, 10∆ide*_Ssuis_*, 10∆ide*_Ssuis_*∇ide*_Ssuis_*_EcoRI, 10∆ide*_Ssuis_*∇ide*_Ssuis_*_C195S were then added at 4 × 10^6^ CFU/ml and the whole incubated at 37°C for 2h. VCP was used at concentrations where it is known to inhibit complement significantly but not completely [19].

### Opsonophagocytosis assay

For opsonophagocytosis assays with purified porcine neutrophils, 1.5 x 10^5^ CFU *S. suis* strain 10, 10∆ide*_Ssuis_*, 10∆ide*_Ssuis_* ∇ide*_Ssuis_*_EcoRI, ∆ide*_Ssuis_*∇ide*_Ssuis_*_C195S were incubated with 100 µl of porcine serum for 0.5h at 37°C on a rotator. Bacteria were added from glycerol stocks prepared at the late exponential growth phase. The chosen experimental serum was drawn from a piglet with meningitis five days after experimental infection with *S. suis* strain 10 within a different animal experiment approved under the permit number TVV 11/16 by the ethics committee of the Landesdirektion Sachsen. This serum has moderate levels of specific anti*-S. suis* IgM (20.4 ELISA units/ml) and relatively low anti*-S. suis* IgG (35.8 ELISA units/ml) as confirmed by anti*-S. suis* IgM and IgG ELISA. Serum of colostrum-deprived piglets (CDS) served as positive control, anti*-S. suis* hyperimmune serum as negative control. Porcine neutrophils were separated as described by Seele et al. 2015 using ficoll density centrifugation [10]. 5 × 10^6^ purified porcine neutrophils were added to the pre-incubated serum containing the above mentioned 1.5 x 10^5^ *S. suis* corresponding to a multiplicity of infection (MOI) of 0.03. Samples were then incubated for 2h at 37°C on a rotator and the survival factor determined as described for the whole blood survival assays above.

### sC5b-9 ELISA

Porcine sC5b-9 was measured by ELISA as described by Messias-Reason et al. [49]. with minor modifications, using a monoclonal antibody against a neoepitope of C5b-9 [50]. Briefly, ELISA plates were coated with a cross-reactive monoclonal anti-C5b-9 antibody, plasma samples were applied at a dilution of 1:3 and detection was performed with a rabbit anti-human cross-reactive C5 IgG primary antibody followed by a peroxidase-conjugated goat anti-rabbit IgG antibody. Zymosan-activated normal porcine serum (set as 1000 ELISA units) was used as standard.

### Animal experimental infection

Thirty-six specific-pathogen free German Landrace piglets known to be free of *sly*^+^ *mrp*^+^ *epf*^+^ *cps2, cps*7 and *cps*9 *S. suis* were divided into four infection groups with nine animals per group. The animals in the respective groups were intranasally challenged with 1.5 x 10^9^ CFU *S. suis* strain 10, 10∆ide*_Ssuis_*, 10∆ide*_Ssuis_* ∇ide*_Ssuis_*_EcoRI, 10∆ide*_Ssuis_*∇ide*_Ssuis_*_C195S at an age of 8 weeks after predisposition with 1% acetic acid as described [46]. Bacterial infection cultures were grown to mid-exponential growth phase. The animal experiment was approved by the Committee on Animal Experiments of the Lower Saxonian State Office for Consumer Protection and Food Safety under the permit number 33.12–42,502-04–16/2305A. Handling and treatment of animals was in strict accordance with the principles of the European Convention for the Protection of Vertebrate Animals Used for Experimental and Other Scientific Purposes as well as the German Animal Protection Law. Animals were clinically monitored including measurement of the inner body temperature, assessment of movements and feed intake every eight hours (piglets were only fed at these time points). Piglets were regarded morbid if the inner body temperature was equal to or greater than 40.2°C. Piglets were euthanized for animal welfare reasons when reaching predefined endpoints such as convulsions or the inability to stand indicating severe meningitis or acute polyarthritis, respectively. Furthermore, piglets were euthanized if high fever (≥ 40.5°C), apathy and anorexia persisted over 36 h. Surviving piglets were euthanized two weeks after infection. Necropsies were performed by a standardized protocol and samples for histopathological as well as bacteriological investigations were taken as described previously [46]. Isolated *S. suis* strains were screened in a MP-PCR for detection of the *cps1*, *cps2, cps7, cps9, mrp, epf, sly, arcA and gdh* [51]. All isolates from the 10∆ide*_Ssuis_* infection group were tested in an ide*_Ssuis_* PCR with the primer pair 800vorEcoRI, 800nachEcoRI to confirm the absence of the ide*_Ssuis_* gene. All isolates from the CSF, the tonsils and chosen inner organs of the 10∆ide*_Ssuis_*∇ide*_Ssuis_* _C195S infection group were subjected to an ide*_Ssuis_* PCR with the primer pair IdeSsuis_con_fo, IdeSsuis_con_rev, followed by sequencing to confirm the presence of the C195S point mutation. All isolates from the 10∆ide*_Ssuis_*∇ide*_Ssuis_* EcoRI infection group as well as all CSF isolates from the wt and 10∆ide*_Ssuis_*∇ide*_Ssuis_* _C195S infection groups were tested in a PCR with the primer pair 800vorEcoRI, 800nachEcoRI. The resulting 1619 bp PCR products weres cut with EcoRI and separated by 1% agarose gel electrophoresis to discriminate between *S. suis* wt and the complemented strain 10∆ide*_Ssuis_*∇ide*_Ssuis_* _ EcoRI.

### Statistical analysis

All experiments were repeated at least three times. Data was analyzed for normal distribution by the Kolmogorov-Smirnov normality test. In case of normal distribution, student’s unpaired, two-tailed t-test was conducted. If data was not normally distributed, the nonparametric two tailed Mann–Whitney test was applied. Kaplan–Meier diagrams representing mortality and morbidity were analyzed with the log-rank test. Concentration dependency was analyzed by calculating the Person correlation coefficient. A confidence interval of 95% was chosen for all analyzes. Figures of all experiments represent the mean and standard deviation. Outliers were calculated by Graphpad QuickCalcs (https://www.graphpad.com/quickcalcs/grubbs1/) and excluded from analysis. Probabilities were considered as follows p < 0.05 *, p < 0.01 **, p < 0.001 ***. Flow cytometric data was analyzed using FlowJo^TM^ _V10 or Flowing Software version 2.5.1.

## Abbreviations

αEry: porcine anti-sheep erythrocyte serum
BCR: B-cell receptor
CDS: colostrum-deprived piglet serum
CFU: Colony forming units
CSF: cerebrospinal fluid
FITC: fluorescein isothiocyanate
IdeE: Immunoglobulin G degrading enzyme of *Streptococcus equi* subsp. *equi*
IdeP: Immunoglobulin G degrading enzyme of *Streptococcus phocae*
IdeS: Immunoglobulin G degrading enzyme of *Streptococcus pyogenes*
Ide*_Ssuis_*: Immunoglobulin M degrading enzyme of *Streptococcus suis*
IdeZ: Immunoglobulin G degrading enzyme of *Streptococcus equi* subsp. *zooepidemicus*
LB-broth: Luria Bertani Broth
MFI: geometric mean fluorescence intensity
MRP: Muramidase-released protein
OPA: Opsonophagocytosis assay
PBS: Phosphate buffered saline
PCR: Polymerase chain reaction
PE: phycoerythrin
POD: horseradish peroxidase
RT: Room temperature
SD: Standard deviation
SF: survival factor
*S. suis*: *Streptococcus suis*
ST: *Streptococcus suis* sequence type
TBST: Tris buffered saline Tween20
THB: Todd-Hewitt Broth
VCP: vaccinia virus complement control protein
wt: wild type

## References

[1] Staats JJ, Feder I, Okwumabua O, et al *Streptococcus suis*: past and present. Vet Res Commun. 1997;21:381–407. 9266659 10.1023/a:1005870317757

[2] Baele M, Chiers K, Devriese LA, et al The Gram-positive tonsillar and nasal flora of piglets before and after weaning. J Appl Microbiol. 2001;91:997–1003. 11851806 10.1046/j.1365-2672.2001.01463.x

[3] Segura M, Calzas C, Grenier D, et al Initial steps of the pathogenesis of the infection caused by *Streptococcus suis*: fighting against nonspecific defenses. FEBS Lett. 2016;590:3772–3799. 27539145 10.1002/1873-3468.12364

[4] Goyette-Desjardins G, Auger J-P, Xu J, et al *Streptococcus suis*, an important pig pathogen and emerging zoonotic agent—an update on the worldwide distribution based on serotyping and sequence typing. Emerg Microbes Infect. 2014;3:e45. 26038745 10.1038/emi.2014.45 PMC4078792

[5] Okura M, Osaki M, Nomoto R, et al Current taxonomical situation of *Streptococcus suis*. Pathogens. 2016;5:45. 10.3390/pathogens5030045 PMC5039425 27348006

[6] Fittipaldi N, Segura M, Grenier D, et al Virulence factors involved in the pathogenesis of the infection caused by the swine pathogen and zoonotic agent *Streptococcus suis*. Futur Microbiol. 2012;7:259–279. 10.2217/fmb.11.149 22324994

[7] Segura M, Fittipaldi N, Calzas C, et al Critical *Streptococcus suis* virulence factors: are they all really critical? Trends Microbiol. 2017;25:585–599. 28274524 10.1016/j.tim.2017.02.005

[8] Lecours M-P, Gottschalk M, Houde M, et al Critical role for *Streptococcus suis* cell wall modifications and suilysin in resistance to complement-dependent killing by dendritic cells. J Infect Dis. 2011;204:919–929. 21849289 10.1093/infdis/jir415

[9] Roy D, Grenier D, Segura M, et al Recruitment of factor H to the *Streptococcus suis* Cell surface is multifactorial. Pathogens. 2016;5:47. 10.3390/pathogens5030047 PMC5039427 27399785

[10] Seele J, Beineke A, Hillermann LM, et al The immunoglobulin M-degrading enzyme of *Streptococcus suis*, IdeSsuis, is involved in complement evasion. Vet Res. 2015;46:1–14. 25928761 10.1186/s13567-015-0171-6 PMC4404118

[11] Seele J, Singpiel A, Spoerry C, et al Identification of a novel host-specific IgM Protease in *Streptococcus suis*. J Bacteriol. 2013;195:930–940. 23243300 10.1128/JB.01875-12 PMC3571317

[12] Rungelrath V, Wohlsein JC, Siebert U, et al Identification of a novel host-specific IgG protease in *Streptococcus phocae* subsp. *phocae*. Vet Microbiol. 2017;201:42–48. 28284621 10.1016/j.vetmic.2017.01.009

[13] Ochsenbein AF, Zinkernagel RM. Natural antibodies and complement link innate and acquired immunity. Immunol Today. 2000;5699:624–630. 10.1016/s0167-5699(00)01754-0 11114423

[14] Tizard IR Veterinary immunology. 9th ed. St. Louis (MO): Elsevier Health Sciences; 2013.

[15] Klimovich VB IgM and its receptors: structural and functional aspects. Biochemistry (Mosc). 2011;76:534–549. 21639833 10.1134/S0006297911050038

[16] Bennett KM, Rooijakkers SHM, Gorham RD Let’s tie the knot: marriage of complement and adaptive immunity in pathogen evasion, for better or worse. Front Microbiol. 2017;8:1–17. 28197139 10.3389/fmicb.2017.00089 PMC5281603

[17] Heyman B, Wigzell H Specific IgM enhances and IgG inhibits the induction of immunological memory in mice. Scand J Immunol. 1985;21:255–266. 3992195 10.1111/j.1365-3083.1985.tb01428.x

[18] Sun J, Butler JE Sequence analysis of pig switch mu, C mu, and C mu m. Immunogenetics. 1997;46:452–460. 9321424 10.1007/s002510050305

[19] Thorgersen EB, Pharo A, Haverson K, et al Inhibition of complement and CD14 attenuates the *Escherichia coli*-induced inflammatory response in porcine whole blood. Infect Immun. 2009;77:725–732. 19047409 10.1128/IAI.01305-08 PMC2632024

[20] Seele J, Hillermann LM, Beineke A, et al The immunoglobulin M-degrading enzyme of *Streptococcus suis*, IdeSsuis, is a highly protective antigen against serotype 2. Vaccine. 2015;33:2207–2212. 25825330 10.1016/j.vaccine.2015.03.047

[21] Lynskey NN, Reglinski M, Calay D, et al Multi-functional mechanisms of immune evasion by the streptococcal complement inhibitor C5a peptidase. PLoS Pathog. 2017;13:1–29. 10.1371/journal.ppat.1006493 PMC5555575 28806402

[22] Buffalo CZ, Bahn-Suh AJ, Hirakis SP, et al Conserved patterns hidden within group A Streptococcus M protein hypervariability recognize human C4b-binding protein. Nat Microbiol. 2016;1:16155. 27595425 10.1038/nmicrobiol.2016.155 PMC5014329

[23] Frick I-M, Rasmussen M, Schmidtchen A, et al SIC, a secreted protein of *Streptococcus pyogenes* that inactivates antibacterial peptides. J Biol Chem. 2003;278(19):16561–16566. 12621031 10.1074/jbc.M301995200

[24] Kraiczy P, Hellwage J, Skerka C, et al Immune evasion of *Borrelia burgdorferi*: mapping of a complement-inhibitor factor H-binding site of BbCRASP-3, a novel member of the Erp protein family. Eur J Immunol. 2003;33:697–707. 12616490 10.1002/eji.200323571

[25] Lathem WW, Bergsbaken T, Welch RA Potentiation of C1 esterase inhibitor by StcE, a metalloprotease secreted by *Escherichia coli* O157:H7. J Exp Med. 2004;199:1077–1087. 15096536 10.1084/jem.20030255 PMC2211892

[26] Li Q, Ma C, Fu Y, et al H specifically capture novel Factor H-binding proteins of *Streptococcus suis* and contribute to the virulence of the bacteria. Microbiol Res. 2017;196:17–25. 28164787 10.1016/j.micres.2016.11.011

[27] Seitz M, Beineke A, Singpiel A, et al Role of Capsule and Suilysin in Mucosal Infection of Complement-Deficient Mice with *Streptococcus suis*. Infect Immun. 2014;82:2460–2471. 24686060 10.1128/IAI.00080-14 PMC4019146

[28] Nordenfelt P, Waldemarson S, Linder A, et al Antibody orientation at bacterial surfaces is related to invasive infection. J Exp Med. 2012;209:2367–2381. 23230002 10.1084/jem.20120325 PMC3526361

[29] Mäkelä O, Rouslahti E, Seppälä IJT Affinity of IgM and IgG antibodies. Immunochemistry. 1970;7:917–932. 4992788 10.1016/0019-2791(70)90053-4

[30] Söderberg JJ, von Pawel-Rammingen U The streptococcal protease IdeS modulates bacterial IgGFc binding and generates 1/2Fc fragments with the ability to prime polymorphonuclear leucocytes. Mol Immunol. 2008;45:3347–3353. 18533265 10.1016/j.molimm.2008.04.013

[31] Brown JS, Hussell T, Gilliland SM, et al The classical pathway is the dominant complement pathway required for innate immunity to *Streptococcus pneumoniae* infection in mice. Proc Natl Acad Sci USA. 2002;99:16969–16974. 12477926 10.1073/pnas.012669199 PMC139253

[32] Kerr AR, Paterson GK, Riboldi-Tunnicliffe A, et al Innate immune defense against pneumococcal pneumonia requires pulmonary complement component C3. Infect Immun. 2005;73:4245–4252. 15972516 10.1128/IAI.73.7.4245-4252.2005 PMC1168602

[33] Paterson GK, Mitchell TJ Innate immunity and the pneumococcus. Microbiology. 2006;152:285–293. 16436416 10.1099/mic.0.28551-0

[34] Bogaert D, Thompson CM, Trzcinski K, et al The role of complement in innate and adaptive immunity to pneumococcal colonization and sepsis in a murine model. Vaccine. 2010;28:681–685. 19892042 10.1016/j.vaccine.2009.10.085 PMC2810519

[35] Okumura CYM, Anderson EL, Döhrmann S, et al IgG protease Mac/IdeS is not essential for phagocyte resistance or mouse virulence of M1T1 group A Streptococcus. MBio. 2013;4:e00499–13 – e00499–13. 10.1128/mBio.00499-13 PMC3735186 23900173

[36] Xiao G, Wu Z, Zhang S, et al Mac Protein is not an Essential Virulence Factor for the Virulent Reference Strain *Streptococcus suis* P1/7. Curr Microbiol. 2017;74(1):90–96. 27847975 10.1007/s00284-016-1160-3

[37] Plebani A, Mira E, Mevio E, et al IgM and IgD concentrations in the serum and secretions of children with selective IgA deficiency. Clin Exp Immunol. 1983;53:689–696. 6616961 PMC1535652

[38] Michaelsen TE, Emilsen S, Sandin RH, et al Human secretory IgM antibodies activate human complement and offer protection at mucosal surface. Scand J Immunol. 2017;85:43–50. 27864913 10.1111/sji.12508

[39] Butler JE, Sun J, Navarro P The swine Ig heavy chain locus has a single JH and no identifiable IgD. Int Immunol. 1996;8:1897–1904. 8982774 10.1093/intimm/8.12.1897

[40] Shima H, Takatsu H, Fukuda S, et al Identification of TOSO/FAIM3 as an Fc receptor for IgM. Int Immunol. 2010;22:149–156. 20042454 10.1093/intimm/dxp121

[41] Smith HE, Damman M, van der Velde J, et al Identification and characterization of the cps locus of *Streptococcus suis* serotype 2: the capsule protects against phagocytosis and is an important virulence factor. Infect Immun. 1999;67:1750–1756. 10085014 10.1128/iai.67.4.1750-1756.1999 PMC96524

[42] de Buhr N, Reuner F, Neumann A, et al Neutrophil extracellular trap formation in the *Streptococcus suis*-infected cerebrospinal fluid compartment. Cell Microbiol. 2017;19:1–16. 10.1111/cmi.12649 27450700

[43] Green MR, Sambrook J Molecular cloning: a laboratory manual. 4th ed. Long Island (NY): Cold Spring Harbor Lab. Press; 2012.

[44] Beineke A, Bennecke K, Neis C, et al Comparative evaluation of virulence and pathology of *Streptococcus suis* serotypes 2 and 9 in experimentally infected growers. Vet Microbiol. 2008;128:423–430. 18068316 10.1016/j.vetmic.2007.10.028

[45] Takamatsu D, Osaki M, Sekizaki T Thermosensitive suicide vectors for gene replacement in *Streptococcus suis*. Plasmid. 2001;46:140–148. 11591139 10.1006/plas.2001.1532

[46] Baums CG, Kaim U, Fulde M, et al Identification of a novel virulence determinant with serum opacification activity in *Streptococcus suis*. Infect Immun. 2006;74:6154–6162. 17057090 10.1128/IAI.00359-06 PMC1695488

[47] Hammerschmidt S, Wolff S, Hocke A, et al Illustration of pneumococcal polysaccharide capsule during adherence and invasion of epithelial cells. Infect Immun. 2005;73:4653–4667. 16040978 10.1128/IAI.73.8.4653-4667.2005 PMC1201225

[48] Baums CG, Kock C, Beineke A, et al *Streptococcus suis* bacterin and subunit vaccine immunogenicities and protective efficacies against serotypes 2 and 9. Clin Vaccine Immunol. 2009;16:200–208. 19109449 10.1128/CVI.00371-08 PMC2643536

[49] Messias-Reason IJ, Hayashi SY, Nisihara RM, et al Complement activation in infective endocarditis: correlation with extracardiac manifestations and prognosis. Clin Exp Immunol. 2002;127:310–315. 11876755 10.1046/j.1365-2249.2002.01772.x PMC1906352

[50] Mollnes TE, Lea T, Frøland SS, et al Quantification of the terminal complement complex in human plasma by an enzyme-linked immunosorbent assay based on monoclonal antibodies against a neoantigen of the complex. Scand J Immunol. 1985;22:197–202. 2412280 10.1111/j.1365-3083.1985.tb01871.x

[51] Silva LMG, Baums CG, Rehm T, et al Virulence-associated gene profiling of *Streptococcus suis* isolates by PCR. Vet Microbiol. 2006;115:117–127. 16431041 10.1016/j.vetmic.2005.12.013

