# Supplementary material for: IgM cleavage by Streptococcus suis reduces IgM bound to the bacterial surface and is a novel complement evasion mechanism
Source: Virulence. 2018 Aug 28;9(1):1314–37. doi: 10.1080/21505594.2018.1496778 (PMC6177247; doi:10.1080/21505594.2018.1496778)
Supplement: Supplemental Material [file kvir-09-01-1496778-s001.docx]

**Supplemental material**

**Table S1** Oligonucleotide primers used in this study.

**Table S2** Antibodies used in Western blot analysis.

**Fig. S1** Coomassie stained SDS-polyacrylamide gel of recombinant His-tagged rIde*_Ssuis_* constructs.

**Fig. S2** Complement hemolysis assays conducted with porcine serum drawn fourteen days post immunization with sheep erythrocytes. (A) Hemolysis assay with different concentrations of rIde*_Ssuis_* and rIde*_Ssuis_*_C195S. (B) Hemolysis assay with different rIde*_Ssuis_* constructs.

**Fig. S3** Anti-Ide*_Ssuis_* Western blot analysis of culture supernatants of *S. suis* wt and mutant strains.

**Fig. S4** EcoRI restriction enzyme digest of a PCR product derived from chromosomal DNA of *S. suis* wt and mutant strains.

**Fig. S5** Southern blot of chromosomal DNA of *S. suis* wt and mutant strains with an ide*_Ssuis_* probe (A) and a pSET5s probe (B).

**Fig. S6** Anti-porcine IgM Western blot analysis with anti-IgM F(ab’)2 and anti-IgM Fc specific antibodies generated in this study.

**Fig. S7** *S. suis* wt shows higher survival factors than the mutant strain 10∆ide*_Ssuis_* and the complemented mutants 10∆ide*_Ssuis_*∇ide*_Ssuis_*_EcoRI and 10∆ide*_Ssuis_*∇ide*_Ssuis_*_C195S in opsonophagocytosis assays.

**Fig. S8** PCR analysis of a 16kb stretch of the *cps2* locus of *S. suis* strain 10 and 10Δide*_Ssuis_*∇ide*_Ssuis_*_EcoRI.

**Fig. S9** Anti-*S. suis* IgM (A), anti-*S. suis* IgG (B) and anti-Ide*_Ssuis_* (C) antibody titers of growing piglets before experimental infection with *S. suis* strain 10, 10∆ide*_Ssuis_*, 10∆ide*_Ssuis_*∇ide*_Ssuis_*_EcoRI and ∆ide*_Ssuis_*∇ide*_Ssuis_*_C195S.

**Table S1** Oligonucleotide primers used in this study

| **primer** | **sequence** | **position^a^** |
| --- | --- | --- |
| preProIdeSPstI | CTTCTGCAGTAAAAGACGCATG | - 504 to - 483 |
| postEndIdeSBamHI | ACGGGATCCTATACTTCAACTTATTCAC | 490 to 517 |
| EcoRI1793ideSsf | GAAAACAAGGATGAATTCGGTTCAGAACTATCAAGAAACATGC | 1776 to 1818 |
| EcoRI1793ideSsr | GCATGTTTCTTGATAGTTCTGAACCGAATTCATCCTTGTTTTC | 1776 to 1818 |
| IdeSsuis_C195S_for | GACCTTAATCTTAGCTTTGCAGCTGTG | 570 to 596 |
| IdeSsuis_C195S_rev  Primer C184S | CACAGCTGCAAAGCTAAGATTAAGGTC  AATGCTTCATTTATTGACCTTAATCTTAGTTTTGCAGCTGTGTCTT | 570 to 596  555 to 600 |
| Sonde 450bp for | GCAAATCAACTAATGTAGAAGCTGC | 2749 to 2773 |
| Sonde 450bp rev | CGTTTCAACTAACTCTGTCTCCTTG | 3164 to 3188 |
| IdeSsuis_con_re | GATTGACACCGCCCTGTGCC | 848 to 867 |
| IdeSsuis_con_fo  800 vor EcoRI  800 nach EcoRI  pSET5sSondefor  pSET5sSonderev  pSET5spräMCSfor  postMCSpSET5s  Orf2_Z_for  Cps2_A_rev  Cps2_A_for  Cps2_E_rev  Cps2_E_for  Cps2_H_rev  Cps2_H_for  Cps2_K_rev | GGGGAAGTAGCGGTAGAGATGAAAG  GCTTCAACACTAGTTGATTCGCTC  CGGTTTGGTGAGGATGTTCGAAC  CGAAAAAAAGAGTTATGATTTCTCTG  GGTTTTTTATAGTGCTTTCCATTTTG  GGCTCGTATGTTGTGTGGAATTGT  CGGTGAAAACCTCTGACACATG  GCTTGGATATTGATCACATGATGG  CCGCCATCTGCGAAAGCAACG  GAAGCGGACGAAGTAAGTCGTCC  CGATGCTATGGTCACCTAGCATTT  GCGTTGATATTAATTCATTCGGTTTTAC  GGCTTCTCTCGCCGTTTCCCAC  GGCGTGGATATGTGGGAAACG  GCTTCTTTTGCTGTTTGCTCAACC | 252 to 276  2564 to 2587  969 to 991  3 to 26  810 to 830  11 to 23  728 to 751  677 to 704  732 to 753  721 to 741  796 to 819 |

a. Numbers indicate the location of the oligonucleotide primer with regard to the initiation

ATG codon. The gene ide*_Ssuis_* ends at position 3426. The ide*_Ssuis_* sequence of *S. suis* P1/7 (Gene ID: 8153996) served as template for the design of primers binding within Ide*_Ssuis_*. The *cps2* sequence of *S. suis* strain 10 (Gene ID: AF118389) served as template for the design of primers binding within the *cps2* locus of *S. suis*.

**TableS2** Antibodies used in Western blot analysis

| Detection of | first antibody | | | |  | | second antibody | | | |  |
| --- | --- | --- | --- | --- | --- | --- | --- | --- | --- | --- | --- |
|  | specificity | source | conjugation | dilution | |  | specificity | source | conjugation | dilution | |
| porcine IgM | anti-pig IgM  (Bethyl Laboratories) | goat^1^ | - | 1:8000 | |  | anti-goat IgG  (Dianova) | rabbit | POD^3^ | 1:5000 | |
| porcine IgM | anti-pig IgM  (Serotec) | mouse^2^ | - | 1:250 | |  | anti-mouse IgG Fc  (Dianova) | goat | POD^3^ | 1:20,000 | |
| porcine IgM | anti-F(ab‘)2 portion of porcine IgM^4^ | rabbit^1^ | - | 1:1000 | |  | anti-rabbit IgG  (Dianova) | goat | POD^3^ | 1:20,000 | |
| porcine IgM | anti-Fc portion of porcine IgM^4^ | goat^1^ | - | 1:1000 | |  | anti-goat IgG  (Dianova) | rabbit | POD^3^ | 1:5000 | |
| Ide*_Ssuis_* | rIde*_Ssuis_* | rabbit^1^ | - | 1:1000 | |  | anti-rabbit IgG  (Dianova) | goat | POD^3^ | 1:20,000 | |

^1^ polyclonal antibody, ^2^ monoclonal antibody, ^3^ peroxidase, ^4^ used for the first time in this study

**
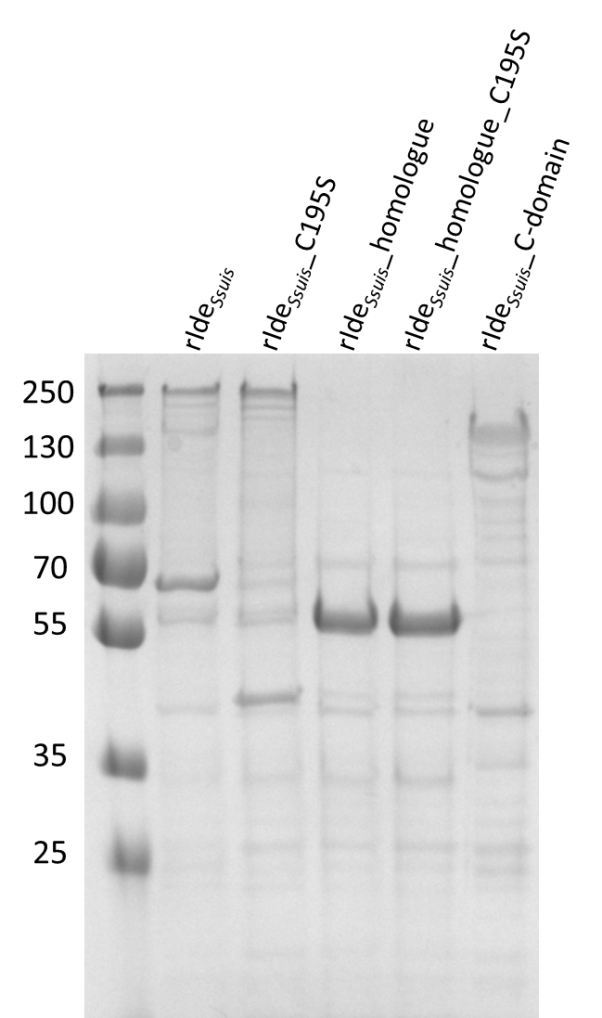
**

**Fig. S1** Coomassie stained SDS-polyacrylamide gel of recombinant His-tagged rIde*_Ssuis_* constructs.

The indicated rIde*_Ssuis_* constructs were separated by SDS-PAGE under reducing conditions in a 10% polyacrylamide gel. Protein concentrations were adjusted prior to loading the gel. Marker bands in kDa are shown in the first lane. The gel was stained by the Coomassie analog Instant Blue^TM^ at room temperature for five hours.

**A**

**
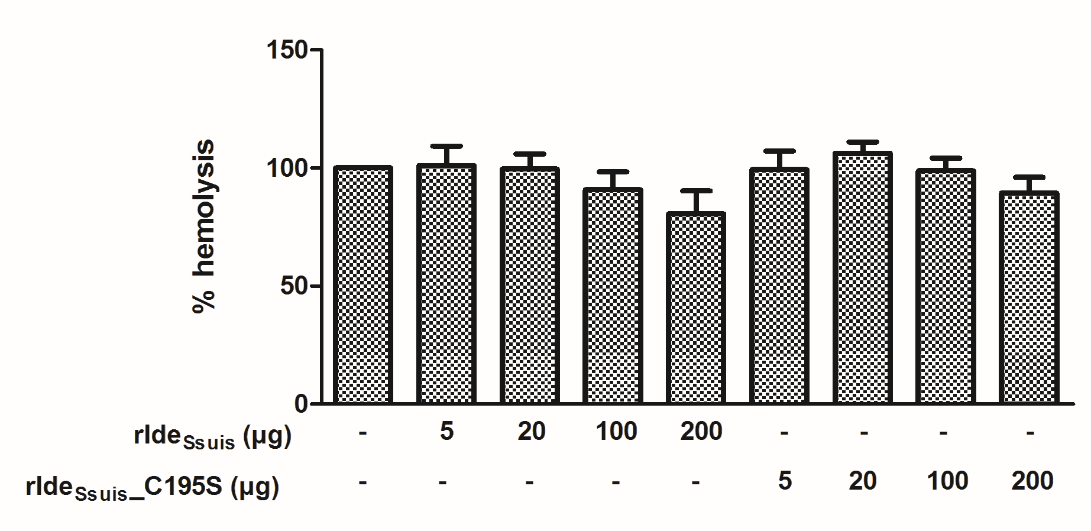
**

**B**

**
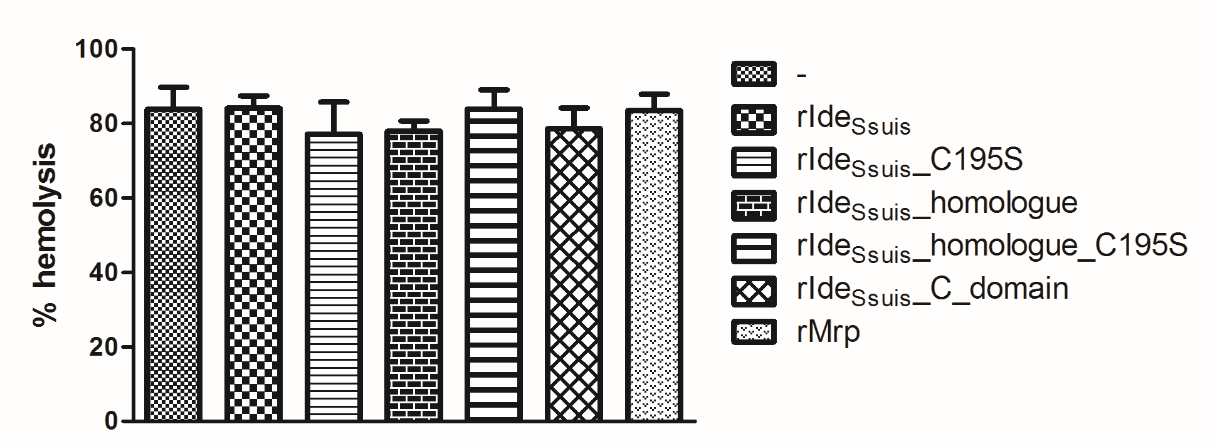
**

**Fig.S2** Neither rIde*_Ssuis_* constructs with IgM cleavage activity nor those without reduced complement mediated hemolysis in the presence of high anti-sheep erythrocyte IgG titers.

Complement hemolysis assays were conducted with porcine serum drawn fourteen days post immunization with sheep erythrocytes. (A) Hemolysis assay with increasing concentrations of rIde*_Ssuis_* and rIde*_Ssuis_*_C195S (n=3). (B) Hemolysis assay with addition of 18µg/ml of the indicated rIde_S_*_suis_* constructs (n=4). One hundred percent hemolysis was defined as the hemolysis induced by water. Recombinant Mrp served as a recombinant control protein and was purified the same way as rIde*_Ssuis_* constructs. Bars and error bars represent mean and standard deviation and significant differences are indicated. Probabilities were considered as follows p<0.05 *, p<0.01 **, p<0.001 ***.


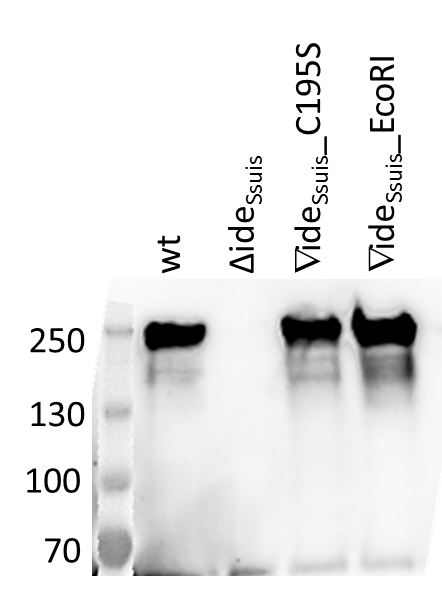


**Fig.S3** Anti Ide*_Ssuis_* Western blot analysis of culture supernatants of *S. suis* wt and mutant strains.

Bacterial supernatants of *S. suis* strain 10 (wt), 10∆ide*_Ssuis_* (∆ide*_Ssuis_*), 10∆ide*_Ssuis_*∇ide*_Ssuis_* _EcoRI (∇ide*_Ssuis_* _EcoRI) and 10∆ide*_Ssuis_*∇ide*_Ssuis_*_C195S (∇ide*_Ssuis_* _C195S) were concentrated 24-fold and subjected to SDS-PAGE under reducing conditions. Detection of Ide*_Ssuis_* in the bacterial supernatants was conducted by Western blotting using a polyclonal rabbit anti-Ide*_Ssuis_* primary antibody and a POD-labeled goat anti-rabbit IgG secondary antibody. Marker bands in kDa are shown on the left hand side.


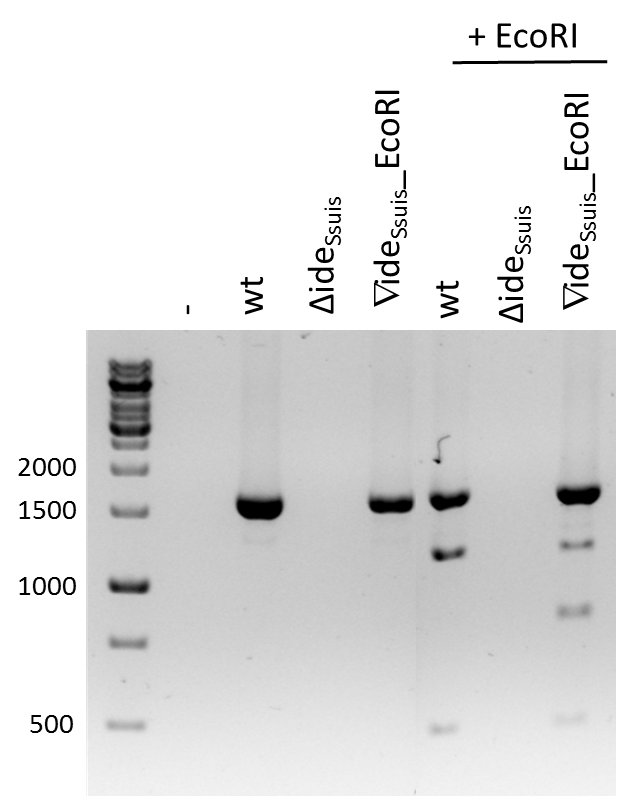


**Fig. S4** EcoRI restriction enzyme digest of a PCR product derived from chromosomal DNA of *S. suis* and mutant strains.

Chromosomal DNA of *S. suis* strain 10 (wt), 10∆ide*_Ssuis_* (∆ide*_Ssuis_*)and 10∆ide*_Ssuis_*∇ide*_Ssuis_* _EcoRI (∇ide*_Ssuis_* _EcoRI) served as template in a PCR with the primers IdeSsuis_con_for and IdeSsuis_con_rev to render a 1619bp amplification product which was subsequently cut with EcoRI and separated by 1% agarose gel electrophoresis. Digest with EcoRI divides the ide*_Ssuis_* amplification product of the wt into two fragments (464bp and 1155bp). The complemented strain 10∆ide*_Ssuis_*∇ide*_Ssuis_* _EcoRI is clearly distinguishable from the wt by an additional expected band at 799bp due to the introduced silent mutation. The sizes of four marker bands in kilo base pairs (kb) are specified on the left side.

**A B**


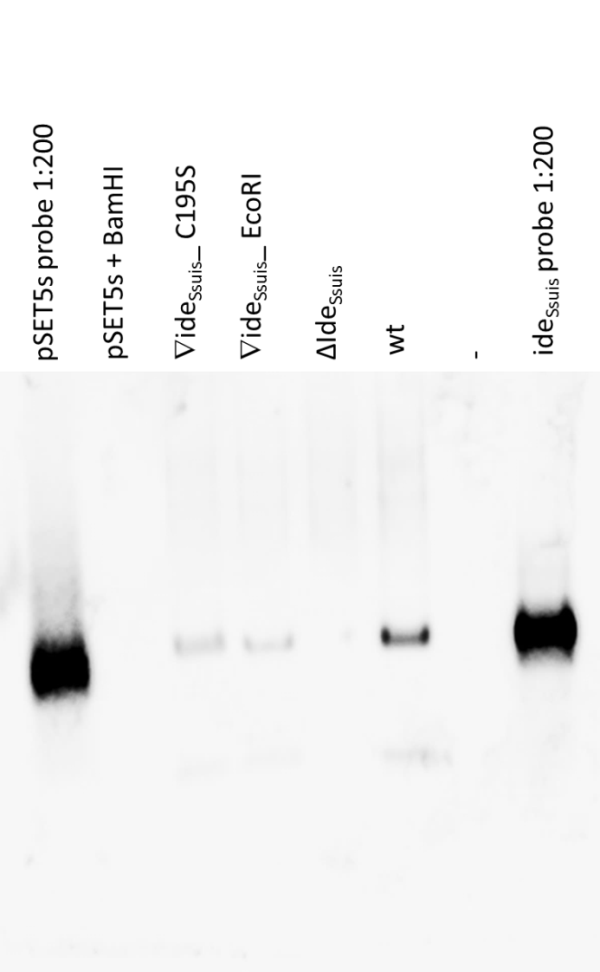
 **
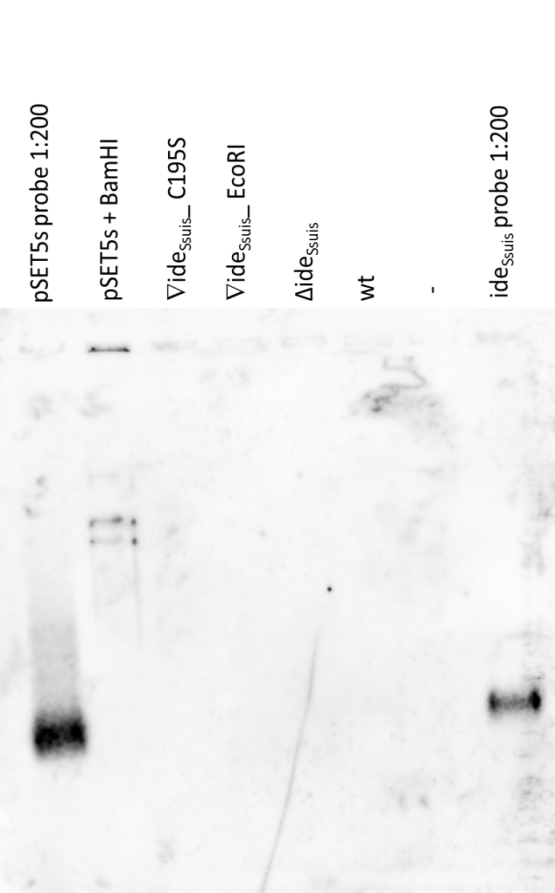
**

**Fig. S5** Southern blot of chromosomal DNA of *S. suis* wt and mutant strains.

Chromosomal DNA of *S. suis* strain 10 (wt), 10Δide*_Ssuis_* (Δide*_Ssuis_*), 10∆ide*_Ssuis_*∇ide*_Ssuis_*_EcoRI (∇ ide*_Ssuis_*_ EcoRI), 10∆ide*_Ssuis_*∇ide*_Ssuis_*_C195S (∇ ide*_Ssuis_*_ C195S) was cut with HincII and blotted onto a positively charged nylon membrane. Hybridization was performed with a biotinylated 450bp probe binding within ide*_Ssuis_* (A) and, after stripping of the membrane, with a biotinylated 412bp probe binding within the pSET5s backbone (B). After hybridization, the membrane was incubated with POD-labeled streptavidin and signals detected by chemiluminescence. For the blot in figure (A) the biotinylated probes served as positive controls and the cut pSET5s vector as negative control. For the blot in figure (B) the biotinylated probes and the cut pSET5s vector served as positive controls.


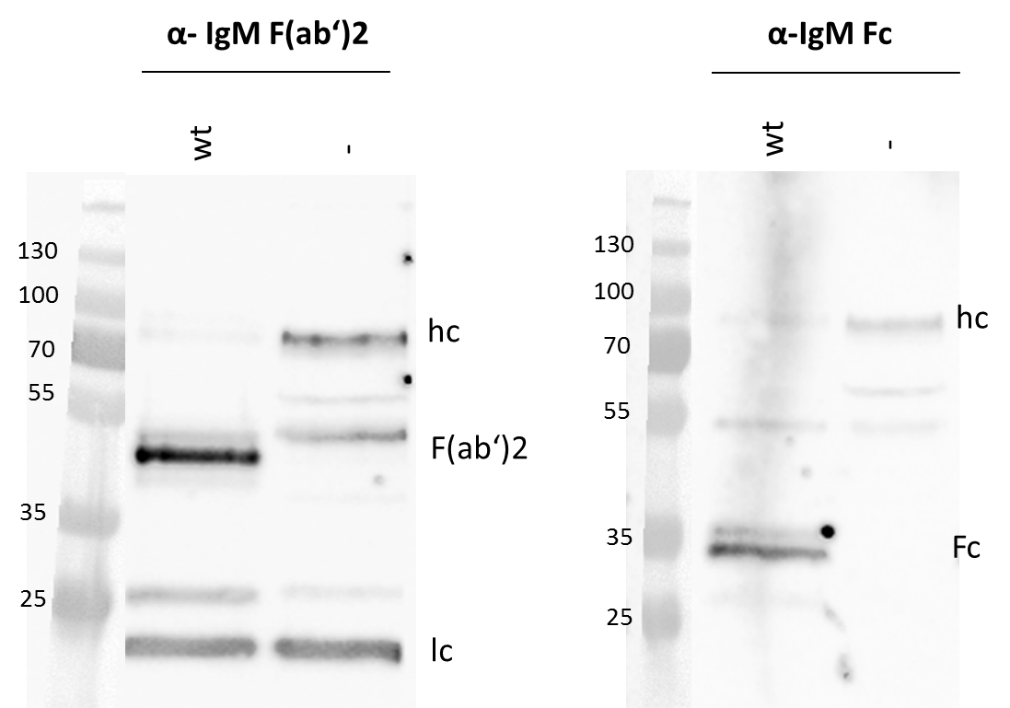


**Fig. S6** Anti-porcine IgM Western blot analysis with anti IgM F(ab’)2 and Fc specific antibodies generated in this study.

Porcine serum was incubated with concentrated supernatant of *S. suis* strain 10 (wt) or phosphate buffered saline (-) and submitted to SDS-PAGE under reducing conditions with a 10% polyacrylamide gel. The different parts of porcine IgM are indicated by the following abbreviations: hc (uncleaved heavy chain), lc (light chain), F(ab’)2 (heavy chain domains V-C1-C2), Fc (heavy chain domains C3-C4). Marker bands in kDa are shown on the left hand side of each blot.

**
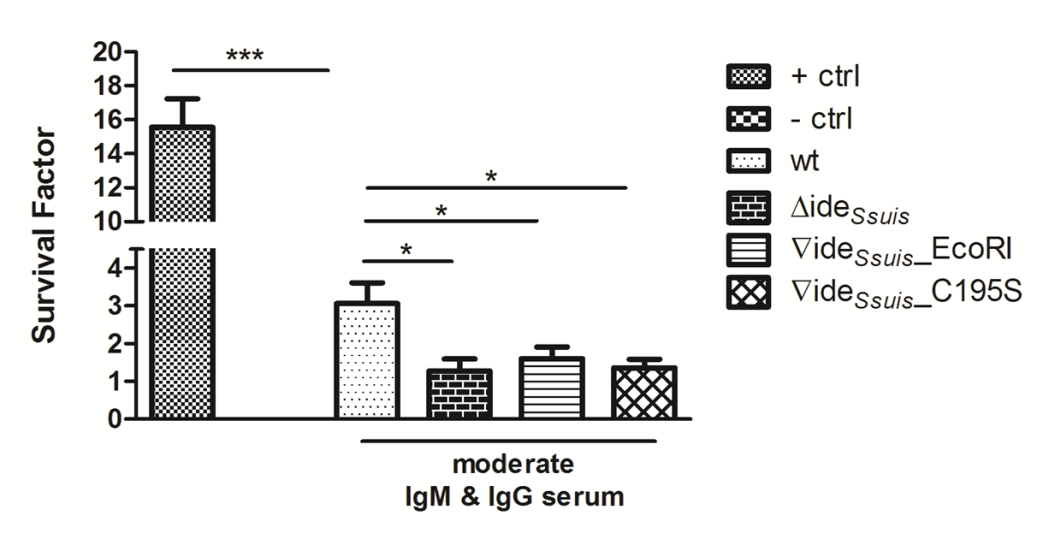
**

**Fig. S7** *S. suis* wt shows higher survival factors than the mutant strain 10∆ide*_Ssuis_* and the complemented mutants 10∆ide*_Ssuis_*∇ide*_Ssuis_*_EcoRI and 10∆ide*_Ssuis_*∇ide*_Ssuis_*_C195S in opsonophagocytosis assays.

Survival of *S. suis* strain 10 (wt), 10∆ide*_Ssuis_* (∆ide*_Ssuis_*), 10∆ide*_Ssuis_* ∇ide*_Ssuis_*_EcoRI

(∇ide*_Ssuis_*_EcoRI), 10∆ide*_Ssuis_*∇ide*_Ssuis_*_C195S (∇ide*_Ssuis_*_C195S) in an opsonophagocytosis

assay (OPA) with purified porcine neutrophils and porcine serum with specific anti *S. suis*

IgM titers (n=7). As a positive control (+ ctrl), *S. suis* strain 10 was incubated with serum of

colostrum deprived piglets (CDS) and as a negative control (- ctrl), *S. suis* strain 10 was

incubated with an anti-*S. suis* hyperimmune serum. Bars and error bars indicate mean and

standard deviation. Significant differences are indicated by asterisks. Probabilities were

considered as follows p<0.05 *, p<0.01 **, p<0.001 ***.

**
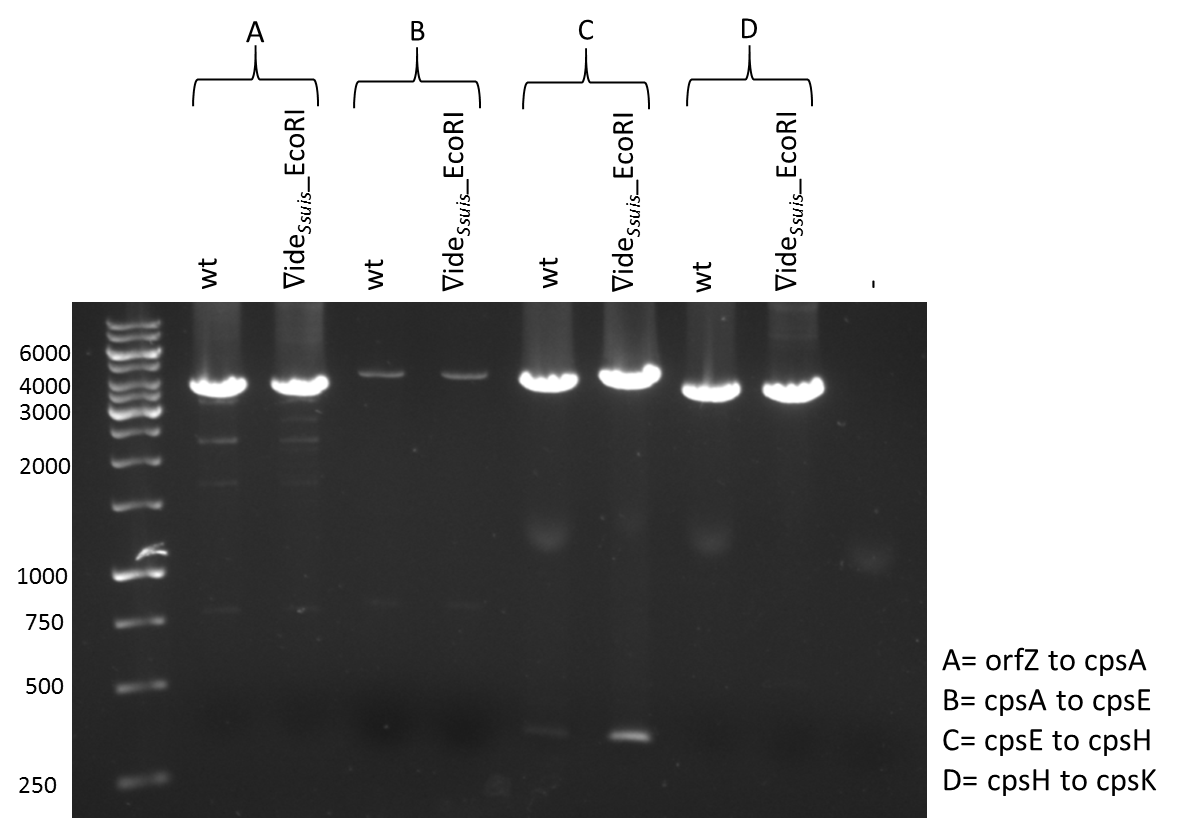
**

**Fig. S8** PCR analysis of a 16kb stretch of the *cps2* locus of *S. suis* strain 10 and 10Δide*_Ssuis_*∇ide*_Ssuis_*_EcoRI.

Chromosomal DNA of *S. suis* strain 10 (wt) and 10Δide*_Ssuis_*∇ide*_Ssuis_*_EcoRI (∇ide*_Ssuis_*_EcoRI) was used as template in four independent PCR reactions each containing a distinct primer pair binding in different regions of the *cps2* locus of *S. suis*. Letters A to D correspond to the indicated regions of the *cps*2 locus of *S. suis*. Each amplicon is approximately 4kb in size. Marker bands in base pairs are depicted in the first lane.

**A**


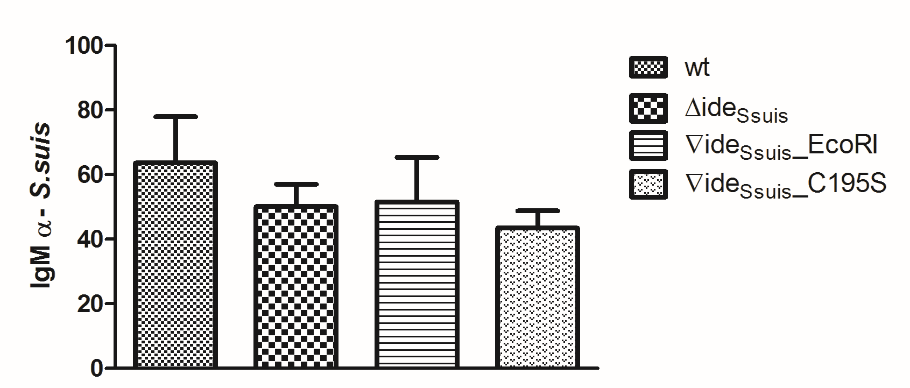


**B**


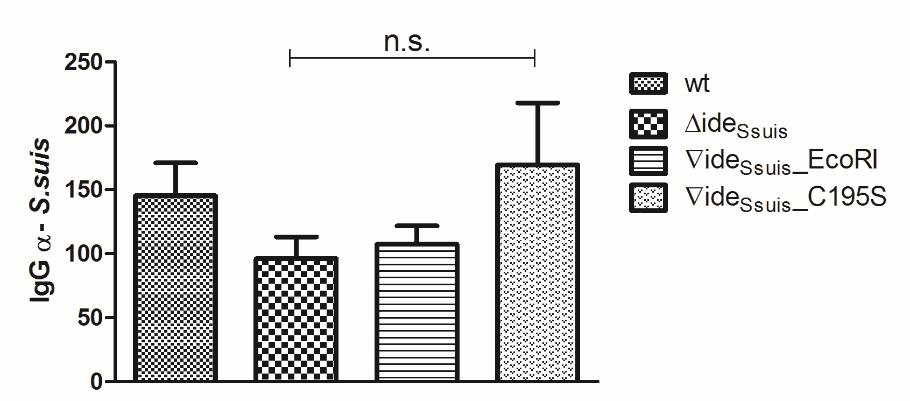


**C**


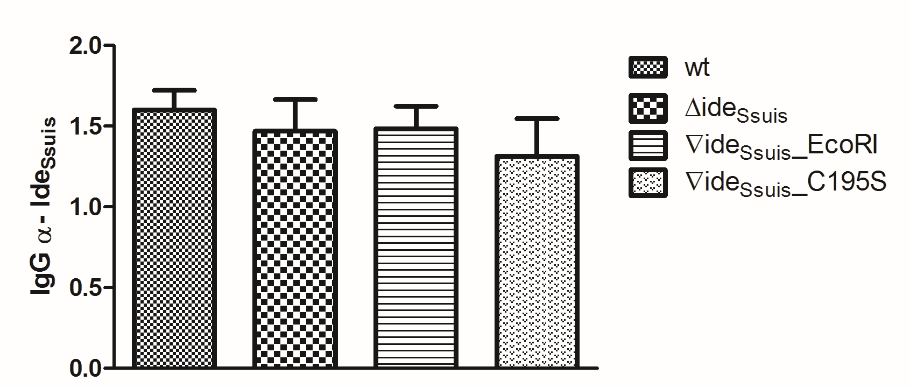


**Fig. S9** Antibody titers of growing piglets before experimental infection with the indicated *S. suis* strains.

Anti *S. suis* IgM (A), anti *S. suis* IgG (B) and anti Ide*_Ssuis_* (C) antibody titers of growing piglets were assessed by ELISA prior to experimental infection with *S. suis* strain 10, 10∆ide*_Ssuis_*(∆ide*_Ssuis_*), 10∆ide*_Ssuis_*∇ide*_Ssuis_*_EcoRI (∇ide*_Ssuis_*_EcoRI) and ∆ide*_Ssuis_*∇ide*_Ssuis_*_C195S (∇ide*_Ssuis_*_C195S). Antibody titers are depicted as relative ELISA Units on the y-axis.
