# Supplementary material for: IgM cleavage by Streptococcus suis reduces IgM bound to the bacterial surface and is a novel complement evasion mechanism
Source: Virulence. 2018 Aug 28;9(1):1314–37. doi: 10.1080/21505594.2018.1496778 (PMC6177247; doi:10.1080/21505594.2018.1496778)
Supplement: Supplemental Material [file kvir-09-01-1496778-s002.docx]

**Supplemental Material intended for revision only**


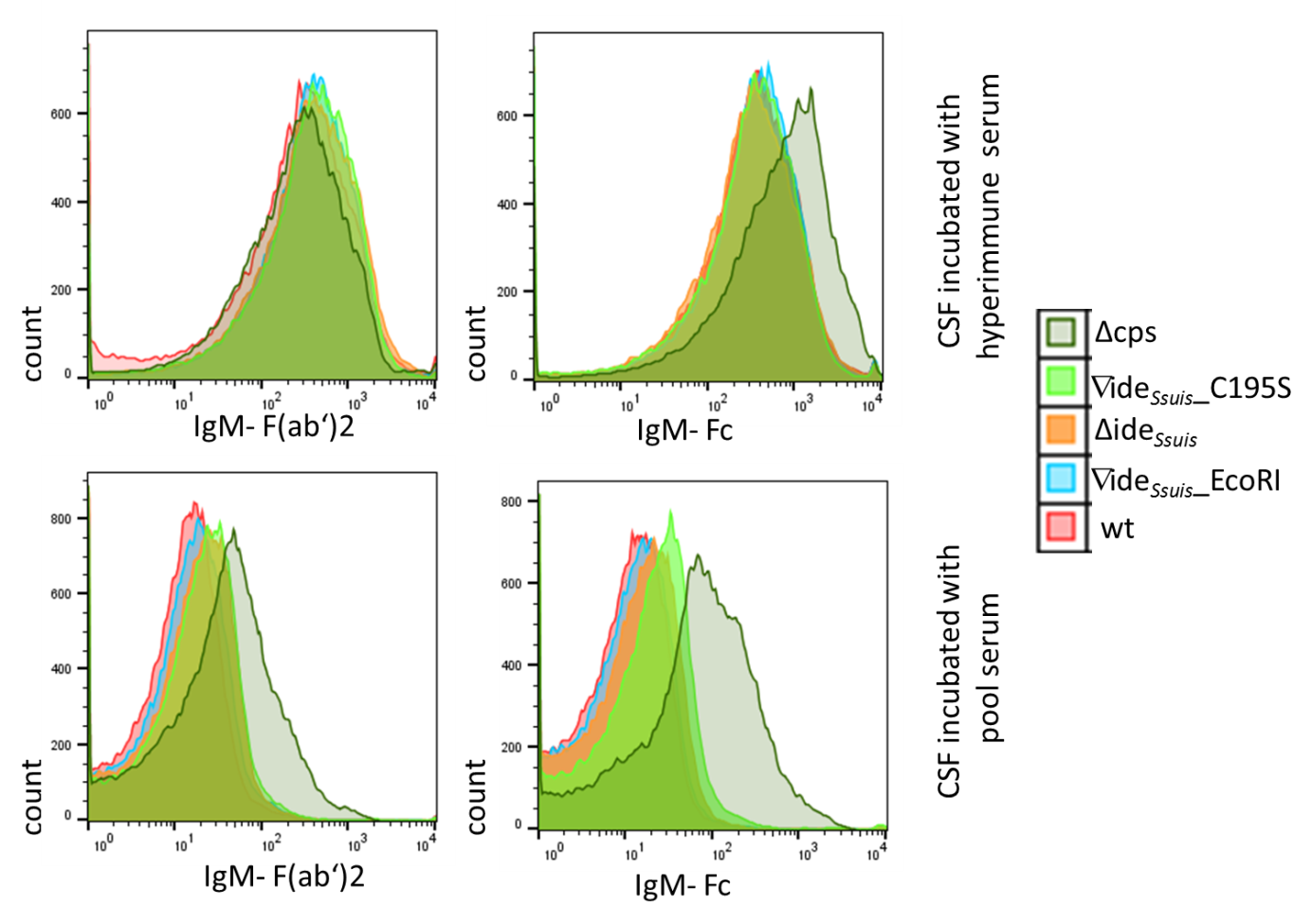


Labeling of *S. suis* strain 10 and mutant strains with IgM F(ab’)2 and IgM Fc antigen after incubation in CSF of healthy piglets supplemented with porcine. 5x10^7^ CFU of *S. suis* 10 (wt), 10∆ide*_Ssuis_* (∆ide*_Ssuis_*), 10∆ide*_Ssuis_*∇ide*_Ssuis_*_EcoRI (∇ide*_Ssuis_*_EcoRI), 10∆ide*_Ssuis_*∇ide*_Ssuis_*_C195S and 10∆cps (∆cps) were incubated in 500µl CSF of healthy piglets supplemented with an anti-*S. suis* strain 10 hyper-immune serum (upper panel) or a pool serum adjusted to contain the same amount of IgM as detected in the CSF of experimentally infected piglets with meningitis (lower panel). Bacteria were stained 1:100 dilutions of rabbit anti-pig IgM F(ab’) and goat anti-pig IgM Fc primary antibodies and donkey anti-rabbit IgG-FITC (1:200) and donkey anti-goat IgG-PE (1:250) secondary antibodies. Labeling with IgM F(ab’)2 antigen is depicted in the upper and lower left panels and labeling with IgM Fc antigen is depicted in the upper and lower right panels. Samples were measured by flow cytometry (BD, FACSCalibur) and analyzed using analyzed using FlowJo^TM^ _V10. The colour legend on the right hand side of the figure applies to all four overlay histograms.
